# Assembly of Human Stem Cell-Derived Cortical Spheroids and Vascular Spheroids to Model 3-D Brain-like Tissues

**DOI:** 10.1038/s41598-019-42439-9

**Published:** 2019-04-12

**Authors:** Liqing Song, Xuegang Yuan, Zachary Jones, Kyle Griffin, Yi Zhou, Teng Ma, Yan Li

**Affiliations:** 1 Department of Chemical and Biomedical Engineering, FAMU-FSU College of Engineering, Florida State University, Tallahassee, FL USA; 2 Department of Biomedical Sciences, College of Medicine, Florida State University, Tallahassee, Florida USA

## Abstract

Human cerebral organoids derived from induced pluripotent stem cells (iPSCs) provide novel tools for recapitulating the cytoarchitecture of human brain and for studying biological mechanisms of neurological disorders. However, the heterotypic interactions of neurovascular units, composed of neurons, pericytes, astrocytes, and brain microvascular endothelial cells, in brain-like tissues are less investigated. The objective of this study is to investigate the impacts of neural spheroids and vascular spheroids interactions on the regional brain-like tissue patterning in cortical spheroids derived from human iPSCs. Hybrid neurovascular spheroids were constructed by fusion of human iPSC-derived cortical neural progenitor cell (iNPC) spheroids, endothelial cell (iEC) spheroids, and the supporting human mesenchymal stem cells (MSCs). Single hybrid spheroids were constructed at different iNPC: iEC: MSC ratios of 4:2:0, 3:2:1 2:2:2, and 1:2:3 in low-attachment 96-well plates. The incorporation of MSCs upregulated the secretion levels of cytokines VEGF-A, PGE2, and TGF-β1 in hybrid spheroid system. In addition, tri-cultured spheroids had high levels of TBR1 (deep cortical layer VI) and Nkx2.1 (ventral cells), and matrix remodeling genes, MMP2 and MMP3, as well as Notch-1, indicating the crucial role of matrix remodeling and cell-cell communications on cortical spheroid and organoid patterning. Moreover, tri-culture system elevated blood-brain barrier gene expression (e.g., GLUT-1), CD31, and tight junction protein ZO1 expression. Treatment with AMD3100, a CXCR4 antagonist, showed the immobilization of MSCs during spheroid fusion, indicating a CXCR4-dependent manner of hMSC migration and homing. This forebrain-like model has potential applications in understanding heterotypic cell-cell interactions and novel drug screening in diseased human brain.

## Introduction

Brain organoids derived from human induced pluripotent stem cells (hiPSCs) emerge as powerful model systems for neurological disease modeling, drug screening, and for studying Zika virus infections^1–5^, which affect over one billion people globally^6^. However, generating brain-region specific organoids with defined structure and function remains a critical challenge because the heterotypic cell-cell interactions to mimic human brain have not yet been fully understood^7–9^. Recently, fusion of human forebrain spheroids of different regions (e.g., human dorsal spheroids with ventral spheroids) has been investigated to model interneuron migration and the interactions of different neuronal subtypes^10–12^. However, the interactions of neuronal cells with other cell types, such as endothelial cells, have not been fully studied in brain organoids^5^.

Neural-vascular interactions, known as neural-vascular unit, play an important role in brain structure and function^13^. It has been suggested that organ-specific endothelial cells secrete a unique set of growth factors that regulate tissue morphogenesis into desired tissue types^14^. Vascular cells can form spheroids to assemble blood vessels or as building blocks for scaffold-free tissue fabrication^15,16^. *In vitro* vascularization of organoids has been attempted for cardiac organoids, showing the enhanced cardiac cell function^17^. *In vivo* vascularization of organoids was realized for the hiPSC-derived organ buds, in which the mixed hiPSC-derived progenitors and endothelial cells efficiently self-organize into functional and vascularized liver or kidney *in vivo* respectively^18,19^. In particular, blood-brain barrier (BBB) is involved in various neurological diseases development, drug administration and nutrient transport^13,20^. Functional BBB models require the interactions of brain microvascular endothelial cells (ECs), astrocytes, neurons, and pericytes, which can be realized using hiPSC-derived cells^21–24^.

Mesenchymal stem cell (MSC)-driven condensation has been observed in organ buds formation based on hiPSC-derived cells for multiple tissue types including kidney, intestine, brain, and heart etc., in the presence of MSCs^19^. Although it remains unclear if MSC-driven condensation is due to adhesion molecules expression or cytoskeleton reorganization, the MSCs support organoid formation from multiple aspects. MSCs reside in virtually all adult tissues including brain and the vicinity of capillaries, and that at least at a subset of MSCs (CD146^+^CD34^−^) can function as pericytes that are closely associated with vasculature^25–27^. When cultured as three dimensional aggregates, MSC secretome are potent source of trophic factors that are modulators of neurogenic niche and could promote angiogenesis and neural differentiation through trophic effects (e.g., fibroblast growth factor (FGF)-2, vascular endothelial growth factor (VEGF), brain-derived neurotrophic factor etc.). MSCs also secrete anti-apoptotic and anti-inflammatory factors, e.g., Prostaglandin E2 (PGE2), and extracellular matrix (ECM) proteins^28^. MSCs displayed higher homing ability to the injuries sites for neural protection, due to the increased expression of CXCR4^29^. Thus, the rationale for the incorporation of ECs and MSCs is to enable the formation of a pro-neurogenic niche that promotes angiogenesis, neo-brain tissue patterning, and maturation.

Our previous studies assembled hiPSC-derived neural progenitor cells (iNPCs) and human bone marrow MSCs in spheroid culture, showing that MSCs promote dorsal cortical spheroid formation^30^. The derivation of cortical spheroids or organoids was also achieved in a suspension bioreactor and from Alzheimer’s patient specific hiPSCs^31–33^. Going one step further, the objective of this study is to investigate heterotypic neural-vascular-mesenchymal interactions in cortical organoids *in vitro* through tri-culture of iNPCs, hiPSC-derived ECs (iECs), and human MSCs. The long-term goal is to fabricate next-generation of brain organoids with additional cellular components from hiPSCs for disease modeling, drug screening, and possibly cell therapy. This study used a simple approach to assemble hiPSC-derived vascular spheroids with hiPSC-derived cortical spheroids in the presence of human MSCs. The cellular localization, fusion kinetics, cytokine secretion and gene expression of brain regional markers, cell-cell interactions, extracellular matrix remodeling proteins, and BBB functional proteins are characterized. This study provides understanding of differential effects of heterotypic cell-cell interactions on hiPSC-based organoid engineering which to date are poorly understood.

## Results

### Aggregate fusion and cell localization of tri-cultured hybrid spheroids

**Figure 1:**
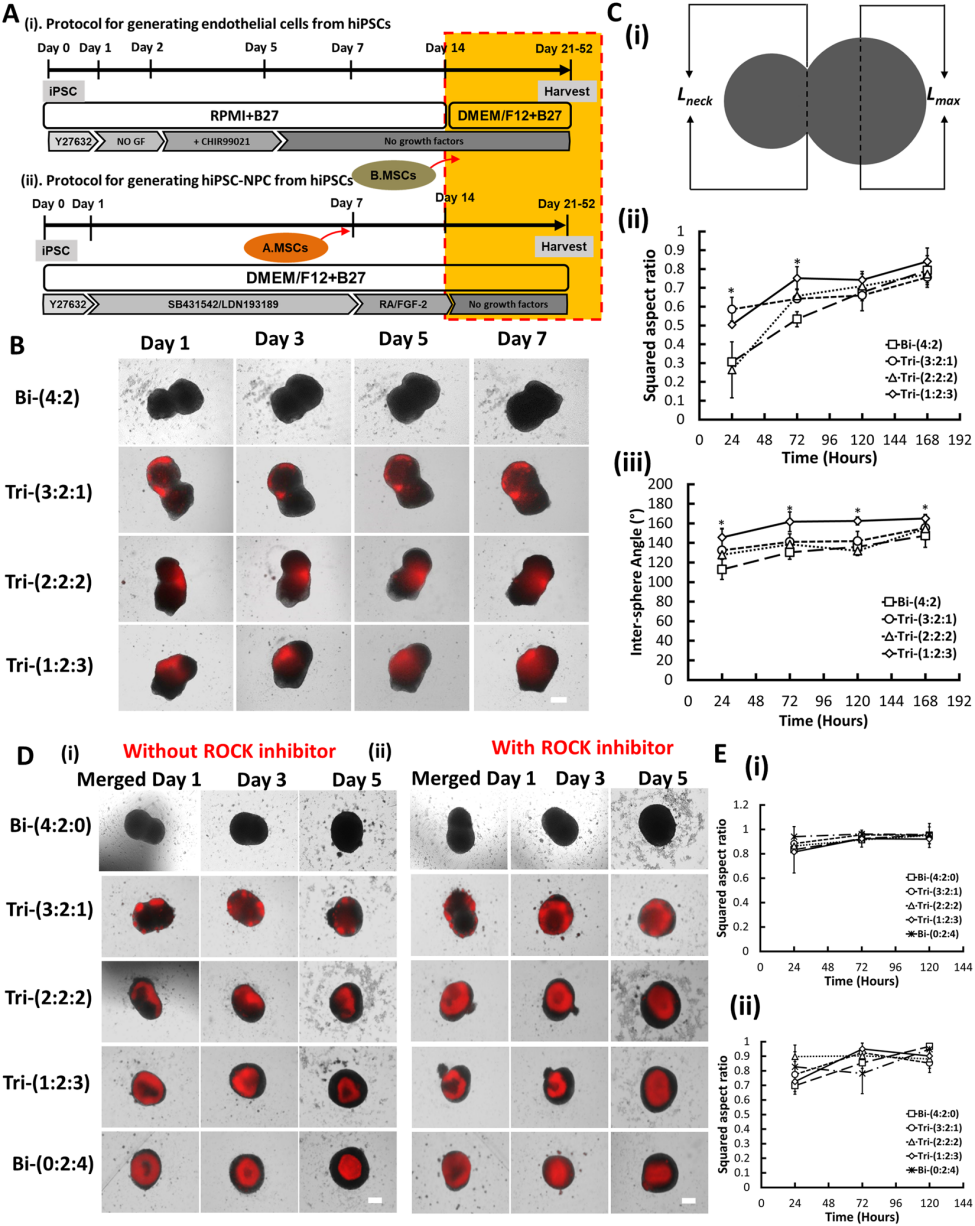
Fusion kinetics of iNPC spheroids and hMSCs with iEC spheroids to construct hybrid spheroids. (**A**) (i) Schematic illustration of endothelial cell (iEC) spheroid derivation from hiPSCs. (ii) Schematic illustration of generating hybrid spheroids from iNPCs. hMSCs were added to iNPCs for iNPC-MSC-iEC spheroids (method A), before iEC transfer. Or hMSCs were added to the well containing iNPC and iEC spheroids for iNPC-iEC-MSC spheroids (method B). hMSCs were labeled with CellTracker Red. (**B**) Phase contrast images of iNPC-iEC-MSC spheroids morphology at day 1, 3, 5, and 7 (total day 15–21). iEC spheroids and MSCs were added to the preformed iNPC aggregates. Scale bar: 400 μm. (**C**) (i) Schematic illustration of calculation of squared aspect ratio of contact length between two aggregates over maximum diameter $(\frac{Lneck}{Lmax})\,{}^{2}$. The aggregation kinetics were evaluated by (ii) the squared aspect ratio over 7 days and (iii) the inter-sphere angle formed by the two aggregates. (**D**) Overlay of phase contrast images (iNPCs and iECs) with fluorescent images (hMSCs) of hybrid spheroids (i) with or (ii) without ROCKi Y27632 (10 μM) when MSCs were added to the culture one week after fusion of iNPC spheroids and iEC spheroids. Scale bar: 400 μm. (**E**) The aggregation kinetics were analyzed in (Ei) and (Eii), respectively. *Indicates *p* < 0.05 for the different test conditions.

The initial optimization of mixing sequence to achieve maximum spheroid fusion for the tri-cultured hybrid spheroids was summarized in Supplementary Materials (Supplementary Information 1 and Figs S1–S4). Briefly, two methods were evaluated: (A) iNPC-MSC-iEC; and (B) iNPC-iEC-MSC (Fig. 1A)^30^. The squared aspect ratio of contact length between the two aggregates over maximum diameter was calculated to assess the spheroid fusion process. The iNPC-MSC spheroids and iEC spheroids, at different iNPC: iEC: MSC ratios of Bi-(4:2), Tri-(3:2:1), Tri-(2:2:2), and Tri-(1:2:3), fused into the aggregates with squared aspect ratio of 0.5~0.7 after 7 days. For iNPC-iEC-MSC spheroids, hMSCs were added into the well of day 14 iNPC-iEC hybrid spheroids and cultured for another 7 days or MSCs and iEC spheroids were added together to the wells containing iNPC spheroids (Fig. 1B–D). hMSCs were integrated with the iNPC-iEC hybrid spheroids and migrated toward the spheroid center. The squared aspect ratio was in the range of 0.9–1.0 (Fig. 1E). The low concentration of ROCKi did not impact the integration of MSCs into iNPC-iEC spheroids. Due to the higher squared aspect ratio (indicating good fusion kinetics), iNPC-iEC-MSC spheroids were mainly used in the following experiments.

The effects of GelTrex (similar to Matrigel, commonly used in organoid formation^3,34^) or hyaluronic acid (HA) (an ECM component in brain^35^) on the fusion of neural spheroids and vascular spheroids was investigated (Supplementary Fig. S5A,B). Quantification of squared aspect ratios over 7 days showed a good fusion tendency for 5% Geltrex (0.7–0.9 by day 7), but not for 10% Geltrex (0.4–0.6 by day 7) (Supplementary Fig. S5A). For HA treatment, 0.025 wt% HA resulted in squared aspect ratio in the range of 0.7~0.9. But 0.05 wt% HA treatment resulted in lower squared aspect ratios (~0.6 by day 7) for Tri-(2:2:2) and Tri-(1:2:3) groups (Supplementary Fig. S5B). The tendency of two spheroids to fuse was disrupted by ROCKi Y27632 at high concentration (Supplementary Fig. S5C). The aspect ratios (0.5–0.7) of 20 μM or 40 μM ROCKi treatment showed a plateau over 7 days. These results indicate that 5% Geltrex and 0.025 wt% HA promote spheroid fusion.

### Cell proliferation, metabolic activity, and cytokine secretion

**Figure 2:**
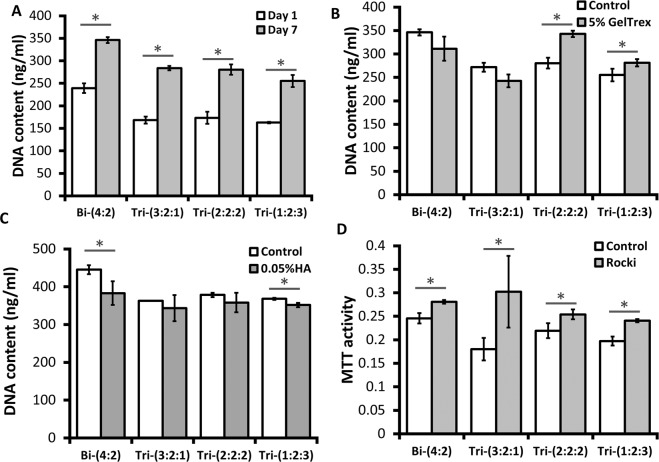
Metabolic activity and DNA content of hybrid spheroids in suspension. (**A**) DNA content of hybrid spheroids after 1 or 7 days of tri-culture (total day 15, 21). (**B**) DNA content of hybrid spheroids with the treatment of 5% Geltrex after 7 days (total day 21) of tri-culture; (**C**) DNA content of hybrid spheroids with the treatment of 0.05 wt% hyaluronic acid (HA) after 7 days (total day 21) of tri-culture. (**D**) MTT activity of hybrid spheroids with the treatment of 20 μM ROCKi Y27632 after 7 days (total day 21) of tri-culture. The control group indicates no treatment. *Indicates *p* < 0.05 for the different test conditions.

DNA content of hybrid spheroids was measured to evaluate the proliferation potential of the hybrid sspheroids. At day 1 after co-culture, Bi-(4:2) group showed the highest DNA content, while the DNA content in the other three group was comparable. After 7 days of co-culture, all four groups showed cell proliferation with increased DNA content (Fig. 2A). Increased DNA content was observed for Tri-(2:2:2) and Tri-(1:2:3) spheroids with the treatment of 5% GelTrex (Fig. 2B). The treatment of 0.05 wt% HA showed no significant difference in DNA content except for Bi-(4:2) spheroids, while 0.025 wt% HA treatment showed no significant difference in DNA content for all the groups (Fig. 2C and Supplementary Fig. S6A). Higher MTT activity was observed for the Bi-(4:2) spheroids compared to the other three groups, and the treatment of ROCKi increased MTT activity (Fig. 2D). 5-Bromo-2′-deoxyuridine (BrdU) assay showed that the cells in S-phase of cell cycle were not homogeneously happening in the spheroids, but tended to be localized at the interface of the spheroids or the spheroid surface (Supplementary Fig. S6B).

**Figure 3:**
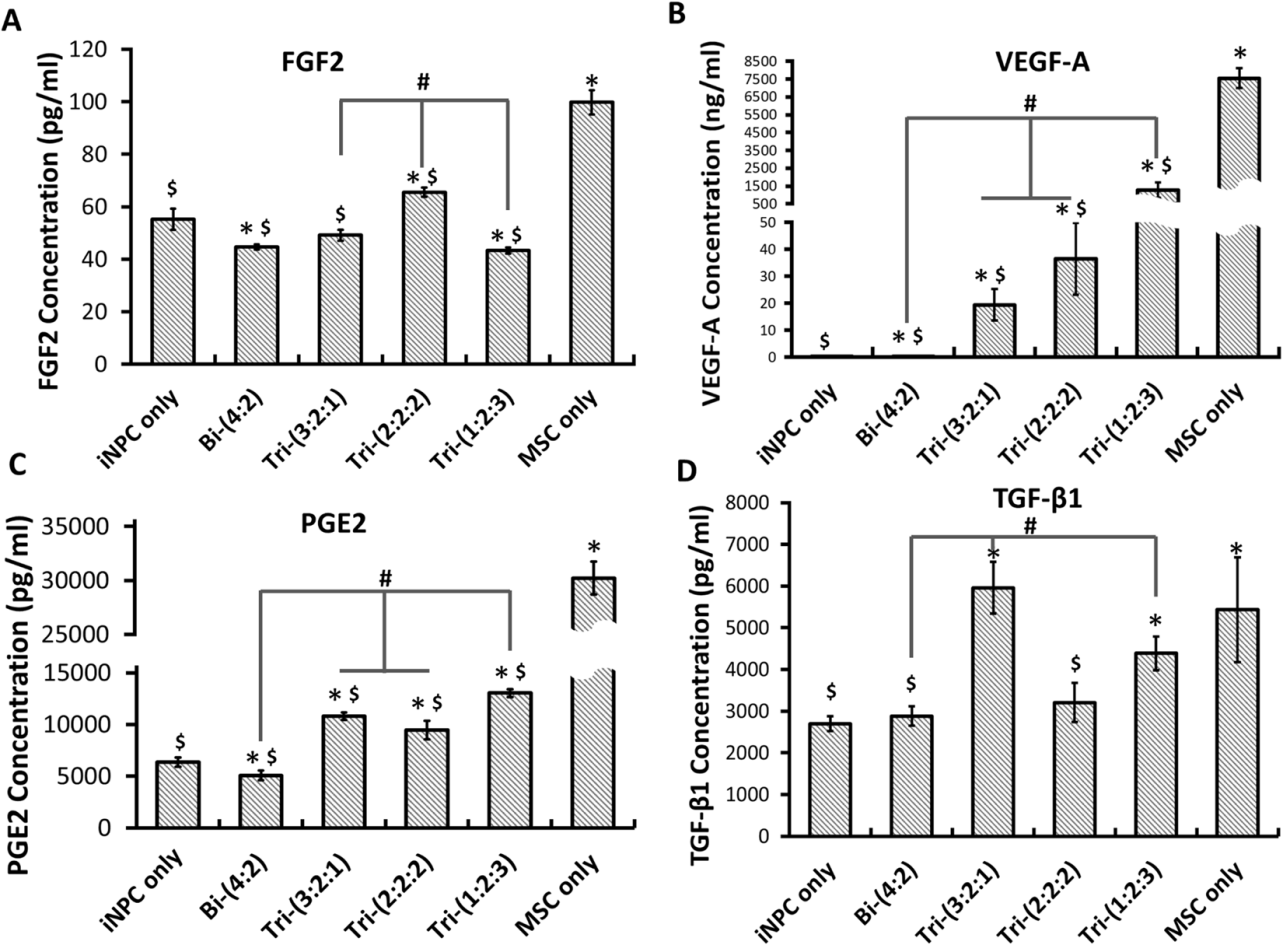
Cytokine secretion by hybrid spheroids during neural differentiation. Culture supernatants were collected and measured by enzyme-linked immunosorbent assay (ELISA) for different spheroids at day 21. Concentrations of (**A**) FGF2, (**B**) VEGF-A, (**C**) PGE2, and (**D**) TGF-β1. iNPC only indicates day 21 iNPC spheroids; MSC only indicates day 7 MSC spheroids. *Indicates *p* < 0.05 for the test conditions compared with the iNPC only control. # indicates *p* < 0.05 among the test conditions. $ indicates *p* < 0.005 for the test conditions compared with the MSC only control. One-way ANOVA for FGF2: P-value < 0.0001, F-value = 115.1; for VEGF-A: P-value < 0.0001, F-value = 126.2; for PGE2: P-value < 0.0001, F-value = 391.2; for TGF-β1: P-value = 0.0002, F-value = 12.3.

The trophic factors secreted by MSCs (e.g., FGF2 and VEGF-A) can enhance the angiogenesis, neurogenesis, and axonal growth during neural tissue regeneration^36,37^. MSCs also secret anti-inflammatory factors, such as TGF-β1 and PEG2, to regulate the immune response^38,39^. The secretion levels of FGF2, VEGF-A, PGE2, and TGF-β1 from hybrid spheroids was characterized (Fig. 3). The highest secretion levels were observed for the hMSC-only group. For FGF2, higher secretion was observed for Tri-(2:2:2) spheroids compared to iNPC-only spheroids (Fig. 3A). The VEGF-A concentration increased with the relative ratio of hMSCs in the hybrid spheroids (Fig. 3B and Supplementary Fig. S7). Similarly, the incorporation of hMSCs in the neural spheroids upregulated PGE2 secretion compared to iNPC-only and Bi-(4:2) groups (Fig. 3C). Tri-(3:2:1) and Tri-(1:2:3) had higher TGF-β1 concentration than the iNPC-only group (Fig. 3D). These results indicate that cytokine secretion from MSCs is maintained in hybrid spheroid culture and the amount is dependent on the ratio of incorporated hMSCs.

### Cortical neural differentiation of tri-cultured hybrid spheroids

**Figure 4:**
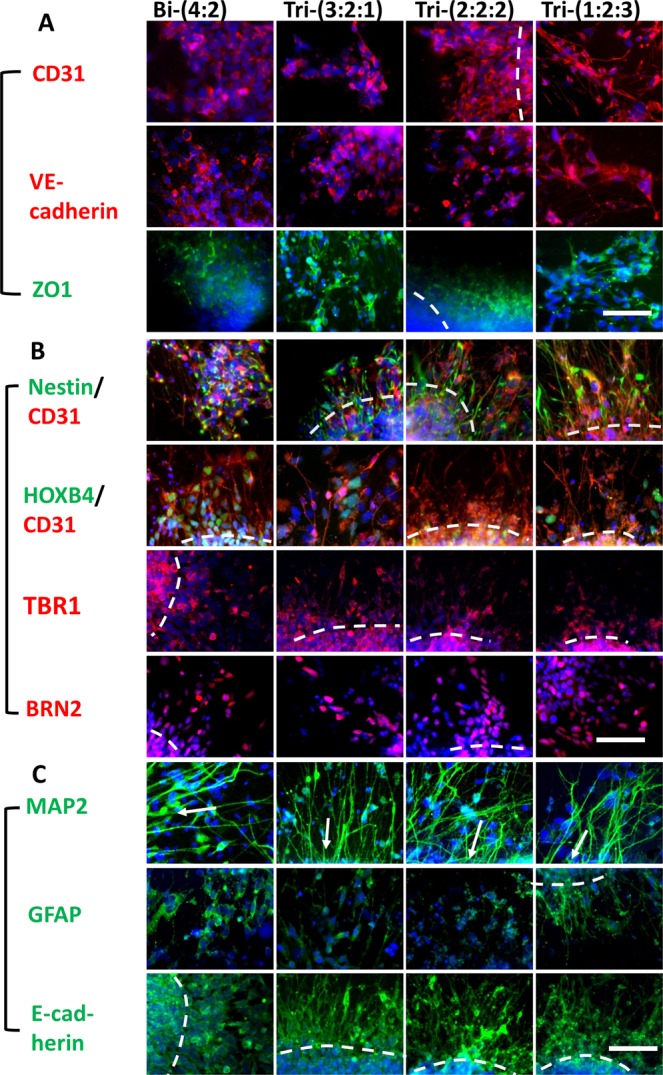
Neural and vascular marker expression of hybrid spheroids. Day 21 hybrid spheroids were replated for three days and immunocytochemistry was performed. (**A**) Representative fluorescent images of vascular markers, including CD31 (red)/Hoechst (blue) for endothelial cells, VE-cadherin (red)/Hoechst (blue) for later stage of endothelial cells, and ZO1 (green)/Hoechst (blue), the tight junction protein expressed by brain microvascular endothelial cells. (**B**) Representative fluorescent images of neural markers, including Nestin (green) for neural progenitors, HOXB4 (green) (a hindbrain marker), TBR1 (red) (forebrain deep cortical layer VI) and BRN2 (red) (forebrain cortical superficial layer II-IV). (**C**) Representative fluorescent images of MAP2 (green) (more mature neurons), GFAP (green) (astrocyte progenitors) and E-cadherin (green) (cell-cell interactions). The dashed lines indicate the locations of spheroids. The arrows point to the direction of spheroid locations. Hoechst: blue. Scale bar: 100 μm.

Hybrid spheroids were replated to investigate their cellular composition (Fig. 4 and Supplementary Fig. S8). The expression of vascular markers, CD31 and VE-cadherin, was observed for all the groups (Fig. 4A). The fused spheroids also expressed ZO1, the tight junction protein of brain microvascular cells. Co-staining of CD31 and Nestin showed that CD31^+^ cells interacted with the Nestin^+^ neural cells (Fig. 4B). The expression of deep cortical layer VI marker TBR1 (and a little cortical layer II-IV BRN2 expression) indicated cortical identity of the hybrid spheroids, although hindbrain marker HOXB4 was also expressed at this stage. Extensive MAP2 expression also showed neuron population, while the expression of GFAP indicated the existence of glial progenitors (Fig. 4C). Additional markers for astrocyte lineage, including S100B, vimentin, and Aldolase C, were also detected (Supplementary Fig. S9). Cells from tri-cultured spheroids had more E-cadherin expression (heterogeneous signal intensity), while Bi-(4:2) group had homogenous expression.

**Figure 5:**
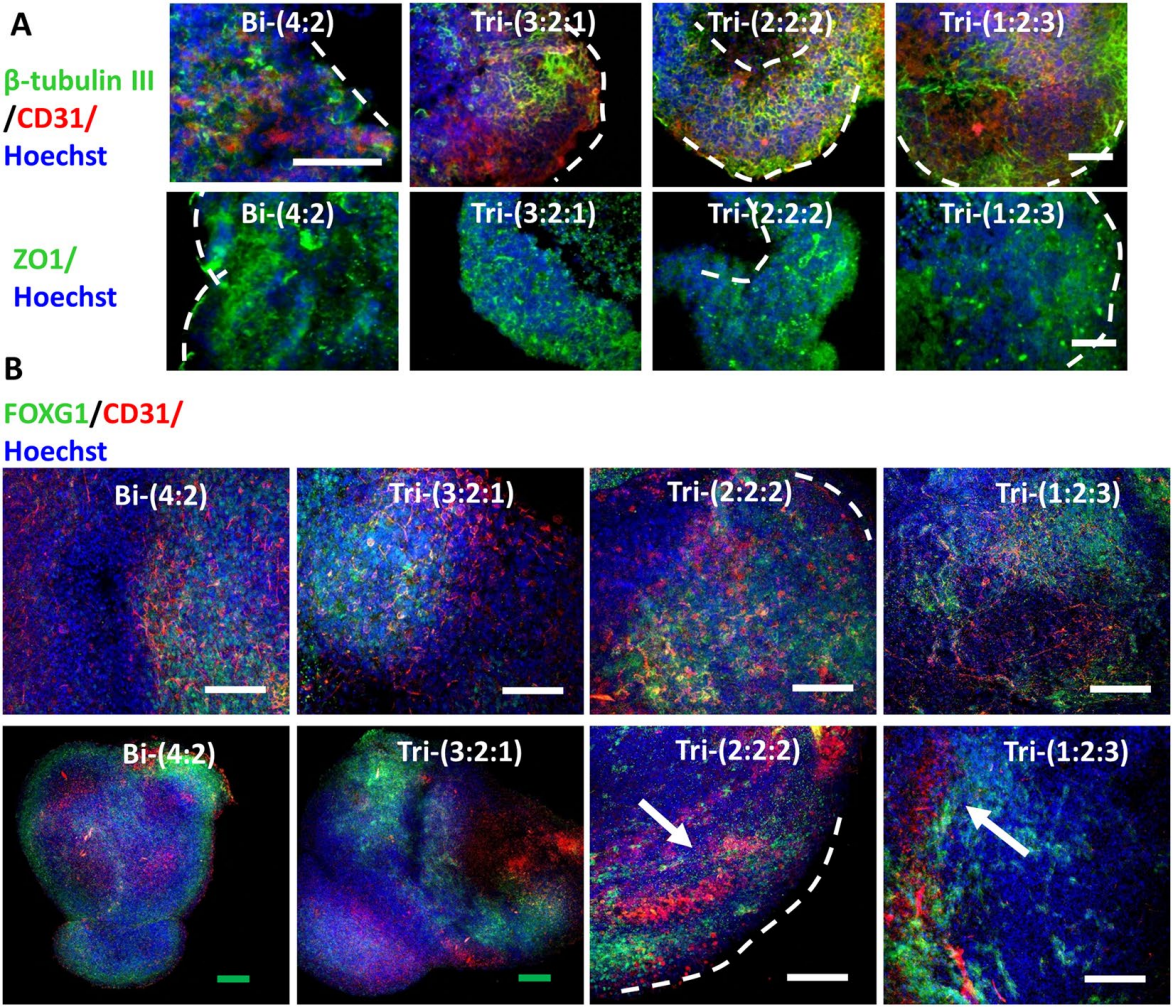
Histology images of early stage hybrid spheroids. (**A**) Images of histological sections of spheroids (total day 21) showed the expression of β-tubulin III (green) and vascular marker CD31 (red), ZO1 (green). Hoechst: blue. The dashed lines indicate the locations of spheroids. Some images show the lumen. Scale bar: 100 μm. (**B**) Confocal images of day 21 spheroids for FOXG1 (green) and CD31 (red). Arrows point to CD31^+^ lumen. Scale bar: 100 μm.

Histological sections were evaluated to assess the *in situ* distribution and localization of iECs and neural cells within the hybrid spheroids (Fig. 5A). Numerous CD31^+^ vascular cells interacted with β-tubulin III^+^ neurons were observed throughout the spheroids (Supplementary Figs S10 and S11). In addition, the distribution of CD31 was more homogenous for Tri-(2:2:2) and Tri-(1:2:3) groups. More ZO1 expression was observed for tri-cultured spheroids. Confocal images of intact spheroids showed the FOXG1^+^ layers and the lumens of CD31^+^ cells (Fig. 5B and Supplementary Fig. S12). The expression of TBR1 inside the fused spheroids was observed, but the expression of BRN2 was minimal at this early stage (Supplementary Fig. S13A). In addition, one side of spheroids expressed HOXB4.

**Figure 6:**
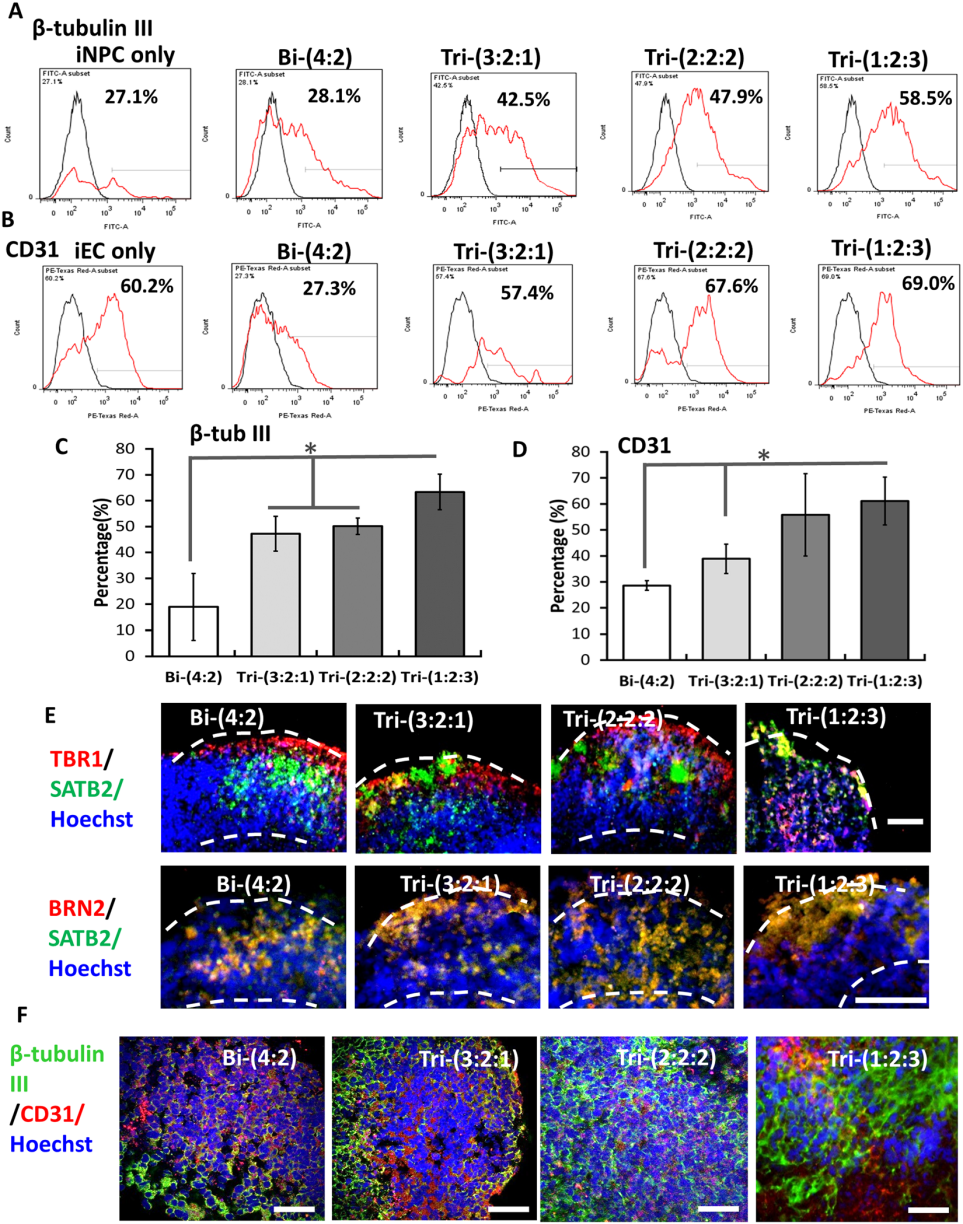
Quantification of neural and vascular markers by flow cytometry and histology images of late stage hybrid spheroids. Flow cytometry histograms for day 21 iEC spheroids, iNPC spheroids, or tri-cultured spheroids for (**A**) β-tubulin III; and (**B**) CD31. Black line: negative control; red line: marker of interest. The percentage indicates the positive cells versus total cells. (**C**) Flow cytometry quantification of β-tubulin III (β-tub III, n = 3) and (**D**) CD31 (n = 3). *Indicates *p* < 0.05 for the different test conditions. (**E**) Images of histological sections of late stage spheroids (total day 47) showed the expression of cortical layer markers: TBR1 (red)/SATB2 (green), BRN2 (red)/SATB2 (green). SATB2: cortical superficial layer II-IV. Hoechst: blue. The dashed lines indicate the locations of spheroids and the cortical layers. Scale bar: 100 μm. (**F**) Confocal images of histological sections of spheroids (total day 47) for β-tubulin III (green) and vascular marker CD31 (red). Scale bar: 50 μm.

The quantification of β-tubulin III showed the higher level for tri-cultured spheroids (i.e., 47.2 ± 6.7%, 50.2 ± 3.2%, 63.4 ± 6.8% for Tri-(3:2:1), Tri-(2:2:2), Tri-(1:2:3) respectively) than Bi-(4:2) group (18.9 ± 12.8%) (Fig. 6A,C). Similarly, higher CD31 expression was observed for the tri-cultured spheroids (i.e., 38.9 ± 5.7%, 55.8 ± 15.8%, 61.1 ± 1.2% for Tri-(3:2:1), Tri-(2:2:2), Tri-(1:2:3) respectively) than Bi-(4:2) group (28.6 ± 1.8%) (Fig. 6B,D). Late stage histology of day 47 forebrain organoids showed the development of superficial cortical layer II-IV indicated by SATB2 and BRN2 expression (Fig. 6E and Supplementary Figs S13B, S14). For Bi-(4:2), SATB2 layer was under the TBR1 layer, while the two layers mixed together for Tri-(3:2:1) and Tri-(2:2:2). For Tri-(1:2:3) (about 80% of spheroids), the TBR1 layer moved toward the center of organoids while the SATB2 layer moved to the surface, according to “inside-out” cortical layer development^32^. The presence of vascular cells (CD31^+^) in late stage forebrain organoids (day 47) was also confirmed in confocal images of histological sections (Fig. 6F and Supplementary Fig. S15). Base membrane proteins Collagen IV and laminin as well as brain matrix chondroitin sulfate proteoglycans and hyaluronic acid were detected (Supplementary Fig. S16). These results indicate that tri-culture promotes neural and vascular differentiation, and the better ratio is Tri-(1:2:3) group.

The electrophysiological properties of the outgrowth cells of the derived spheroids/organoids were examined via patch clamping. As cells within the dense core of the spheroid cannot be visualized by phase contrast microscopy while in the recording chamber, outgrowth cells toward the boundary of the spheroid were chosen for these experiments. Recorded cells displayed fast inward currents and long-lasting outward currents during voltage-clamp recording, suggesting the presence of functional voltage-gated Na^+^ and K^+^ channels, respectively (Supplementary Fig. S17). In addition, a subpopulation of the cells fired rebound action potentials in response to hyperpolarizing current injection during current clamp recording. Spontaneous postsynaptic currents were observed in the absence of stimulation during continuous voltage clamp recording. Cellular morphology was stereotypically neuron-like, with small cell bodies and extensive long and thin projections. Together, these results suggest that the hybrid spheroids have the functional and morphological properties of neurons including synaptic activity.

### Gene expression for brain regional markers, matrix remodeling, and BBB properties

**Figure 7:**
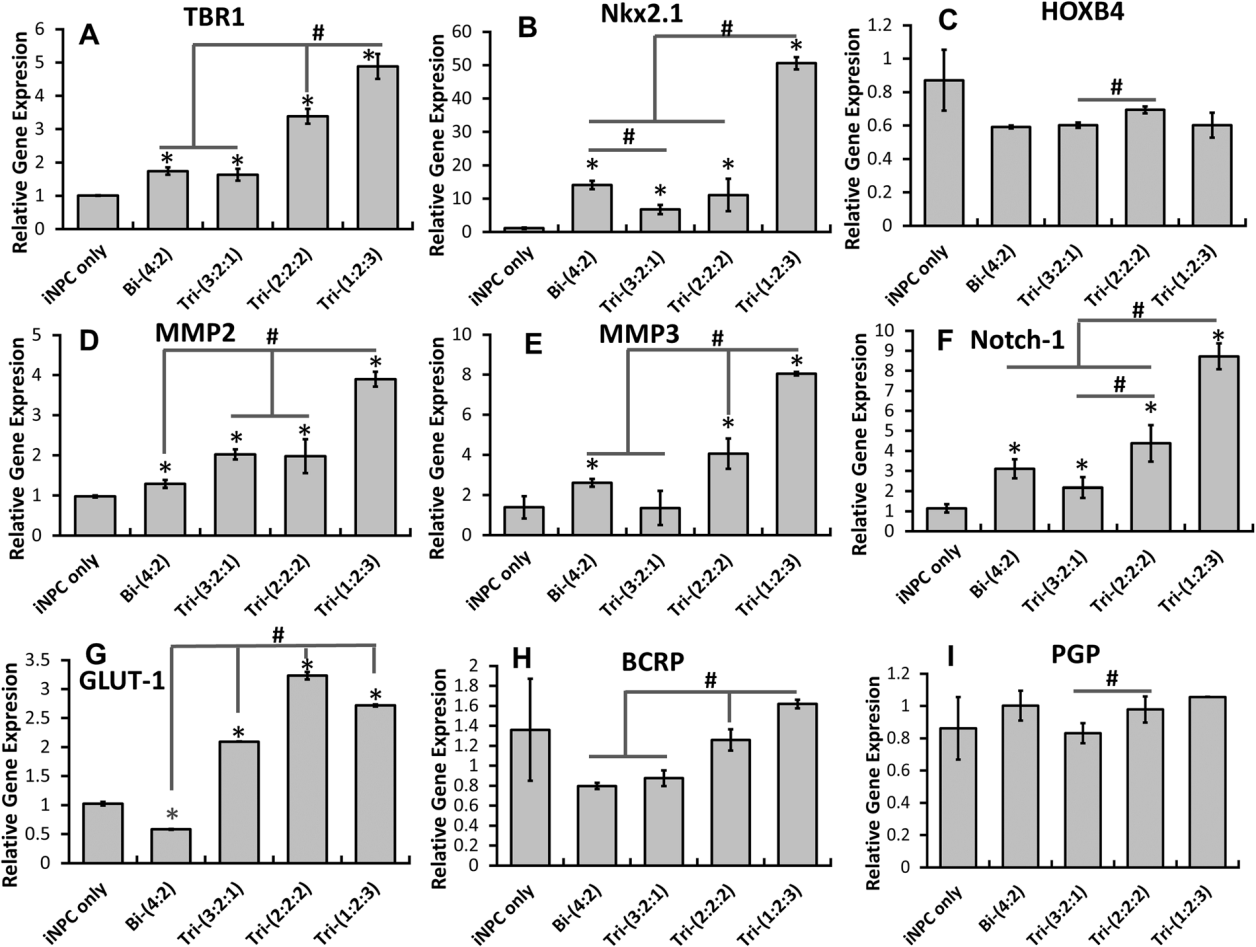
RT-PCR analysis of gene expression of hybrid spheroids. mRNAs were isolated from day 21 hybrid spheroids for RT-PCR. Brain regional marker genes (contribution from iNPCs): (**A**) TBR1, (**B**) Nkx2.1; (**C**) HOXB4. Matrix remodeling and cell-cell communication genes (contribution from MSCs): (**D**) MMP2; (**E**) MMP3; (**F**) Notch-1. Blood-brain barrier-related genes (contributions from iECs): (**G**); GLUT-1; (**H**) BCRP; (**I**) PGP. *Indicates *p* < 0.05 for the test conditions compared with the iNPC only control. # indicates *p* < 0.05 among the test conditions.

Gene expression of brain regional markers TBR1, HOXB4, and Nkx2.1 were determined for day 21 hybrid spheroids (Fig. 7 and Supplementary Fig. S18). The expression level of cortical deep layer VI marker TBR1 increased with the abundance of hMSCs, i.e., 1.74 ± 0.11, 1.63 ± 0.17, 3.39 ± 0.22, 4.88 ± 0.38 fold for Bi-(4:2), Tri-(3:2:1), Tri-(2:2:2), Tri-(1:2:3) respectively (Fig. 7A). Significantly higher expression of Nkx2.1, a ventral regional marker, was observed for hybrid spheroids, i.e., 14.1 ± 1.3, 6.7 ± 1.4, 11.1 ± 4.8, 50.5 ± 1.8 fold for Bi-(4:2), Tri-(3:2:1), Tri-(2:2:2), Tri-(1:2:3) respectively than iNPC-only spheroids (Fig. 7B). However, HOXB4 was similarly expressed for all groups (Fig. 7C).

Matrix metalloproteinases (MMPs) play a critical role in neural cell proliferation, migration and differentiation^40–42^. Significantly higher expression of MMP2 was observed for hybrid spheroids than the iNPC only spheroids, i.e., 1.28 ± 0.10, 2.02 ± 0.13, 1.96 ± 0.43, 3.90 ± 0.19 fold for Bi-(4:2), Tri-(3:2:1), Tri-(2:2:2), Tri-(1:2:3) respectively (Fig. 7D). For MMP3, higher expression was observed for hybrid spheroids than iNPC only spheroids except for Tri-(3:2:1), i.e., 2.60 ± 0.19, 1.36 ± 0.85, 4.06 ± 0.76, 8.05 ± 0.09 fold for Bi-(4:2), Tri-(3:2:1), Tri-(2:2:2), Tri-(1:2:3) respectively (Fig. 7E). Notch signaling is known to enhance cell-cell communications and involved in the BBB formation. Upregulation of Notch-1 expression was observed in the hybrid spheroids, i.e., 3.10 ± 0.48, 2.17 ± 0.51, 4.38 ± 0.91, 8.72 ± 0.65 fold for Bi-(4:2), Tri-(3:2:1), Tri-(2:2:2), Tri-(1:2:3) respectively (Fig. 7F). These results suggest that incorporation of MSCs upregulated MMP2, MMP3, and Notch-1 expression.

The expression of glucose transporter 1 (GLUT-1) and efflux transporters, BCRP and PGP, was determined to demonstrate whether the iECs possess BBB properties. For GLUT-1, higher expression was observed for all tri-cultured groups compared to iNPC only spheroids and Bi-(4:2) group, i.e., 0.58 ± 0.01, 2.10 ± 0.004, 3.23 ± 0.07, 2.72 ± 0.02 fold for Bi-(4:2), Tri-(3:2:1), Tri-(2:2:2), Tri-(1:2:3) respectively (Fig. 7G). BCRP was dependent on the abundance of the hMSCs, and the Tri-(1:2:3) group showed the highest BCRP gene expression, i.e., 0.80 ± 0.03, 0.87 ± 0.08, 1.26 ± 0.10, 1.62 ± 0.04 fold for Bi-(4:2), Tri-(3:2:1), Tri-(2:2:2), Tri-(1:2:3) respectively (Fig. 7H). For PGP, higher expression was observed for Tri-(2:2:2) and Tri-(1:2:3) groups compared to Tri-(3:2:1) group, i.e., 1.00 ± 0.09, 0.83 ± 0.06, 0.98 ± 0.08, 1.06 ± 0.00 fold for Bi-(4:2), Tri-(3:2:1), Tri-(2:2:2), Tri-(1:2:3) respectively (Fig. 7I). These observations indicate that co-culturing iECs with iNPCs and hMSCs increased the expression of GLUT-1 and BCRP.

### Neurite extension and AMD3100 treatment (functional properties)

The influence of GelTrex, HA, and Y-27632 on the neurite morphology was examined (Supplementary Figs S19, S20A, S20B). The neurite outgrowth was enhanced by Y27632, showing more β-tubulin III^+^ axons and dendrites. Geltrex promoted the neurite outgrowth, but the packing density of axons was not high. Prolonged cortical differentiation was performed for day 39 hybrid spheroids treated with Geltrex or 0.025 wt% HA (Supplementary Fig. S20C). The hybrid spheroids contained GABAergic neurons and glutamatergic neurons, and expressed pre-synaptic marker synapsin I and post-synaptic marker PSD95.

**Figure 8:**
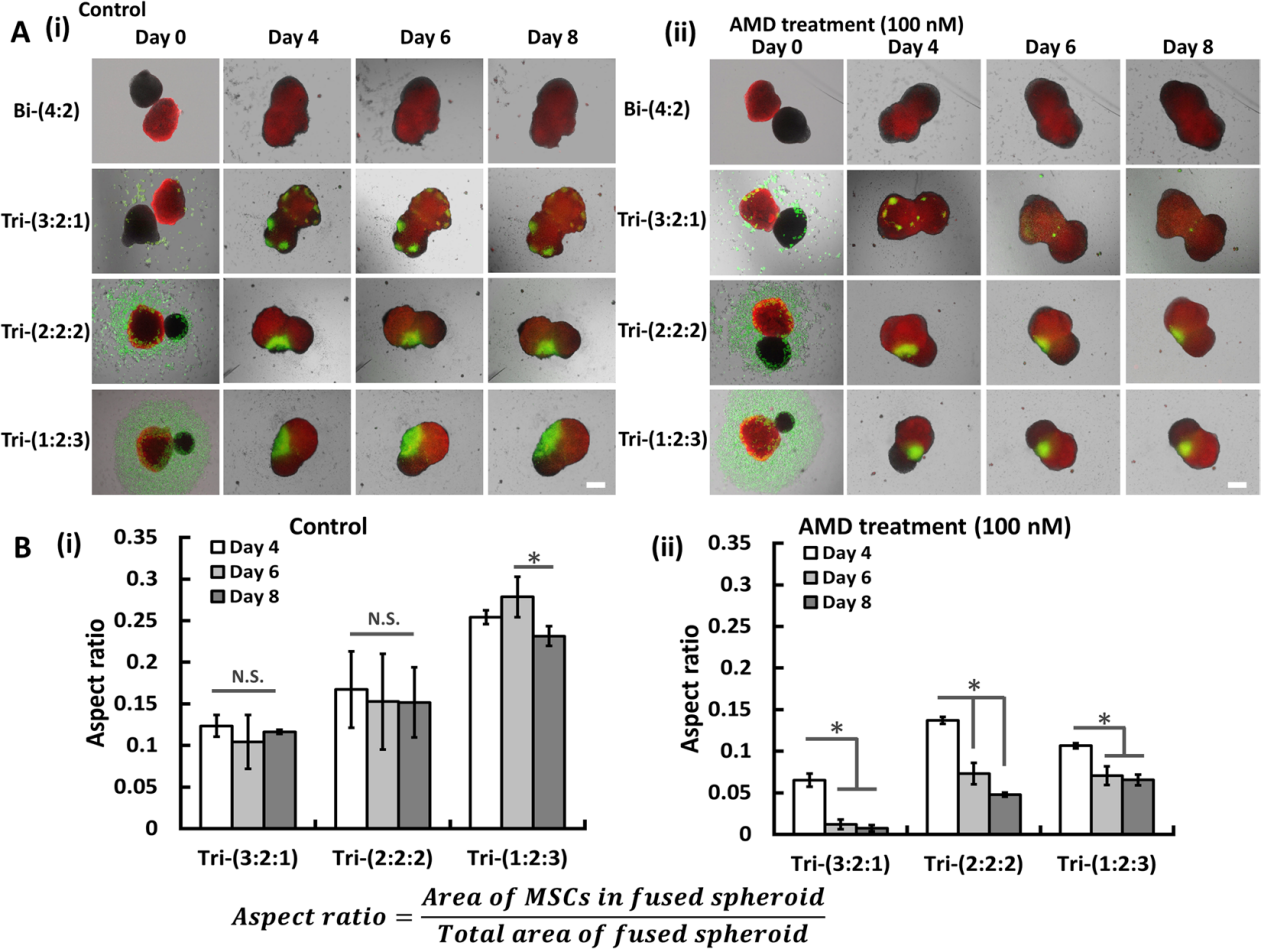
Effects of AMD3100 treatment on the aggregation kinetics of hybrid spheroids. (**A**) Overlay of phase contrast images (iNPCs) with fluorescent images (hMSCs labeled with CellTracker Green, iECs labeled with CellTracker Red) of different hybrid spheroids were either (Ai) untreated (control) or (Aii) treated with CXCR4 inhibitor (AMD3100). Scale bar: 400 μm. (**B**) Analysis of aspect ratios, i.e., area of MSCs in fused spheroids over total area of fused spheroid, for groups either (Bi) untreated (control) or (Bii) treated with CXCR4 inhibitor (AMD3100). *Indicates *p* < 0.05 for the different test conditions.

It has been reported that cell migration in cerebral organoids depends on CXCR4 (a cell homing receptor) activity^12^. To understand the mechanisms of spheroid fusion and the self-sorting behavior of different cell types, the effects of CXCR4 antagonist AMD3100 was investigated (Fig. 8). The MTT activity indicated that AMD3100 treatment had little influence on cell proliferation (Supplementary Fig. S21). iEC spheroids (with CellTracker Red) gradually fused with the iNPC spheroids. Small hMSC (with CellTracker Green) areas sparsely spread over the fused spheroids for Tri-(3:2:1) group. For Tri-(2:2:2) and Tri-(1:2:3) groups, one large area of hMSCs occupied the interface of iEC and iNPC spheroids (Fig. 8Ai,Bi). For AMD3100 treatment, the area occupied by hMSCs was smaller than the control groups (Fig. 8Aii,Bii). Analysis of the relative ratio of area occupied by hMSCs to the total area of fused spheroids showed that the aspect ratio of hMSCs decreased from day 4 to day 8 with AMD3100 treatment (Fig. 8Bii). These results indicate that hMSC migration and invasion into the iEC and iNPC spheroids may be mediated by a CXCR4-dependent manner.

## Discussion

Current hiPSC-derived brain organoids show promising results in modeling different neurological diseases such as microcephaly^3^, lissencephaly^43^, and ZIKV infections^2^. However, the lack of interactions with other cell types such as endothelial cells in current brain organoids model limits their applications^5,9^. As neurological diseases such as BBB breakdown and dysfunction in Alzheimer’s and stroke involve multiple cell types, *in vitro* models such as brain organoids must include relevant cell types to better reconstruct cellular microenvironment^13^. As vascular system is an essential component of brain tissue, incorporating neural-vascular interactions in forebrain organoids is an important step in developing brain organoids *in vitro*.

This study applied a neural-vascular co-patterning method through the fusion of independently derived cortical spheroids and isogenic endothelial spheroids from hiPSCs to introduce vascular cells into the cortical spheroids. To our knowledge, it is the first study to introduce vasculature into the cortical spheroids/organoids through spheroid fusion. Most existing study directly mixed the endothelial cells with the other cell types for 3-D co-culture systems^17,19,44^. The advantage of spheroid fusion method over direct mixing method is that (1) it avoids cell dissociation and re-association process, which could lose many cells; (2) the hybrid spheroid structure can be pre-controlled with special compartment arrangements.

Spheroid fusion method has been used for assembly of dorsal and ventral forebrain spheroids to study interneuron migration and the assembly of iPSC-derived endothelial progenitor spheroids and smooth muscle progenitor spheroids^10,16^. Fusion of spheroids of different cell types is most likely driven by minimization of interfacial free energy and cellular thermodynamics, differential cellular adhesions (e.g., E-cadherin expression), or cortical tension redistribution^28,45,46^. The fusion kinetics was found to be affected by ROCK inhibitor, ECMs in the medium, and mixing sequence based on our study. The addition of high concentration of ROCKi Y-27632 during the initial aggregation delayed fusion process, indicating that cortical tension regulates spheroid fusion process, and actomyosin may play a key role in spheroid fusion^47,48^. The presence of Geltrex and HA (an ECM component in the brain) at an appropriate concentration promotes spheroid fusion. Since MSCs condense into the center of the hybrid spheroids, adding MSCs to iNPC spheroids before iEC spheroid fusion (iNPC-MSC-iEC) would constrict MSCs as the iNPC spheroid core. So the better mixing sequence is iNPC and iEC spheroid fusion in the presence of MSCs (iNPC-iEC-MSC).

### Neural-vascular interactions through spheroid fusion

The results of this study indicate that assembly of vascular spheroids and cortical spheroids enhanced the glucose transporter, GLUT-1 (specifically expressed in endothelial cells in brain^13^), and a polarized efflux transporter BCBP. Structurally, the tight junction protein ZO1 was promoted in the tri-culture, indicating that neurovascular co-patterning promotes the specification of iECs toward brain microvascular cells. Human iPSC-based 2-D co-culture systems of multiple cell types (i.e., neurons, astrocytes, pericytes and brain microvascular endothelial cells) have been recently reported to mimic BBB function with higher trans-endothelial electrical resistance (TEER) properties and study drug permeability *in vitro*^21–24^. Neural-vascular interactions result in the special structure and function of BBB. However, the 3-D BBB models have not been well established due to the complex BBB feature and the difficulty to form micro-vessels structure in 3-D. Some studies use hollow fiber system with perfusion culture or artificially creating microchannels for 3-D vascularization^20^. Although our system is not yet an accurate and perfusable 3-D BBB model, the system recapitulates the anatomical features of the BBB using human stem cells. The inclusion and characterization of additional cell types (e.g., astrocytes), complex 3-D capillary network development (need novel biomaterials design), and perfusion flow study with bioreactors or microfluidics may be explored in future^49^.

Neural-vascular interactions also impact brain tissue patterning. Our results showed the elevated β-tubulin III and CD31 expression, as well as higher TBR1 and Nkx2.1 gene expression in tri-culture, in particular Tri-(1:2:3) group. In addition, the “inside-out” development of cortical superficial layer and deep layer in forebrain is faster for Tri-(1:2:3) group compared to other groups. It has been reported that neural differentiation of hPSCs requires direct association with vascular cells^50^ as well as interactions with hMSCs, possibly through mitogen-activated protein kinase (MAPK) and PI3K-Akt signaling (involved FGF2)^51^. In particular, our tri-culture system promotes the expression of Notch-1, the key protein in Notch signaling which is responsible for cell-cell contact interactions involved ECs^52,53^. In addition, activation of Notch signaling can promote the neural stem cell self-renewal, glial cell differentiation, and neuron regeneration^54,55^. The results in this study indicate that direct contact among iNPCs, iECs, and hMSCs at a given ratio accelerates the development of 3-D cortical tissue structure containing vascular cells to model human brain development.

### The role of MSC in neural-vascular interactions-insoluble ECMs

The interactions of hMSCs and iNPC have been discussed in our previous study^30^. In this study, the presence of hMSCs in addition to iECs enriches ECM localization and affects matrix remodeling. As brain ECMs have limited fibril ECMs such as collagens^20^, the expression of collagen IV in hybrid spheroids was mainly attributed to the incorporation of hMSCs. The elevated MMP-2 and MMP-3 expression indicates the active matrix remodeling, which is required for maintaining the function of neural stem cells^42^. The formation of hMSC aggregates upregulates several types of MMPs (MMP-2, -9, and -1/13)^56^, which were reported to enhance neuronal differentiation through NF-кB signaling^57^. ECM remolding may also be an important contributing factor involved in the migration and invasion of the hMSCs. In this study, the immobilized hMSCs after CXCR4 (receptor for CXCL12/stromal-derived factor-1 chemokine) inhibition reveals that the migration of hMSCs contributes to the fusion of the hybrid spheroids.

### The role of MSC in neural-vascular interactions-soluble cytokines

Secretion of cytokines and neurotrophin is a critical function of hMSCs, which can enhance neurogenesis of hiPSCs^58^. The influence of TGF-β1 and PGE2 secreted by hMSCs on hiPSC-neural differentiation was discussed in our previous study^30^. The upregulated TGF-β1 and PGE2 secretion by hMSCs promotes Nestin and β-tubulin III expression. In this study, two additional growth factors FGF2 (mitogen) and VEGF-A were measured. Our results showed that the main source of VEGF-A is hMSCs, although vascular cells also contribute to VEGF-A secretion. Previous studies have suggested that brain vascular ECs promote neural cell functionality, such as synaptic activities, via the modulation of VEGF signaling and the VEGF receptors were activated by neural cell-secreted nitric oxide^59,60^. In our study, iECs may be a minor source of VEGF-Aas shown in Bi-(4:2) group, as they are not mature enough compared to the bone marrow-derived hMSCs. The elevated VEGF-A in tri-culture (in particular Tri-(1:2:3) group) regulated by Notch signaling is a result of close cell-cell contacts of neural-vascular-mesenchymal cells through autocrine, paracrine, and juxtacrine interactions^61,62^. It was noted that the amount of cytokine produced did not correlate with the DNA content increase in different hybrid spheroids (Fig. 3A), since the DNA content increase was mainly attributed to iNPCs and iECs, but not hMSCs.

The cellular ratio indicates that Tri-(1:2:3) group better promotes neural-vascular interactions than the other groups, indicated by higher cytokine secretion, neural patterning marker expression, BBB-related gene expression, and the cortical layer separation. Consistently, the brain composition is reported to have neuron-to-astrocyte ratio at 1:3^22^, indicating the importance of accessory cells on neural functions. Long-term culture of hMSCs (DNA degradation was observed) is limited in this study using neural differentiation medium in the tri-culture for brain tissue development. Similarly, endothelial cell maturation medium (e.g., EGM-2 medium) was not able to be used to promote mature vascular structure. These results indicate the needs to optimize medium formulations that support all cell types in the co-culture system. In addition, the apoptosis may exist due to upregulated caspase3/7 expression and altered mitochondria bioenergetics on 3-D MSC aggregation due to compaction^63^. An alternative is the use of iPSC-derived MSC (iMSCs) in the tri-culture system as reported by Gao *et al*.^64^.

Vascularization is crucial in the development of brain organoids *in vitro* but remains a significant challenge. To date, the actual vascularization was only achieved *in vivo*^18,19,65^. *In vitro* vascularization needs the accurate design of ECM amounts, the ECM structure (insoluble), and the soluble secreted factors. Recently, colleagues at UC Davis embedded whole brain organoids derived from hiPSCs in Matrigel with isogenic ECs or coated day 34 spheroids with ECs to achieve *in vitro* and *in vivo* vascularization^66^. Another study from Mansour *et al*. (2018) vascularized brain organoids *in vivo* showing the integration of microglia and the functional neuronal networks and blood vessels^65^. All these studies indicate that neural-vascular interactions are indispensable for modeling neurological diseases and screening drugs that require 3-D brain tissue structure^44^. This is in particularly true for the use of diseased hiPSC lines such as Alzheimer’s disease^31^. Development of Alzheimer’s-patient derived cortical organoids containing vascular cells would be important to recapitulate neurodegenerative microenvironment and investigate the cellular response to drug treatments.

## Conclusions

This study assembled hiPSC-derived cortical spheroids and isogenic vascular spheroids in the presence of hMSCs to study neurovascular interactions. The presence of hMSCs promotes cortical neural differentiation, layer separation, cytokine secretion, and cell-cell communication. The presence of iECs provides the BBB-related properties inside the cortical spheroids/organoids. Our results indicate that the elevated Notch signaling, matrix remodeling proteins, and the secretion of VEGF-A through the assembly of cortical spheroids, vascular spheroids, and mesenchymal cells contribute to the accelerated cortical tissue development. This study provides insights to heterotypic cell-cell interactions and potential strategy for the fabrication of next generation of forebrain organoids.

## Methods

### Undifferentiated hiPSC culture

Human iPSK3 cells were derived from human foreskin fibroblasts transfected with plasmid DNA encoding reprogramming factors OCT4, NANOG, SOX2 and LIN28 (kindly provided by Dr. Stephen Duncan, Medical College of Wisconsin, and Dr. David Gilbert, Department of Biological Sciences of Florida State University)^67,68^. Human iPSK3 cells were maintained in mTeSR serum-free medium (StemCell Technologies, Inc., Vancouver, Canada) on 6-well plates coated with growth factor reduced Geltrex (Life Technologies). The cells were passaged by Accutase dissociation every 5–6 days and seeded at 1 × 10^6^ cells per well of 6-well plate in the presence of 10 μM Y27632 (Sigma) for the first 24 hours^33,69,70^.

### Human MSC (hMSC) culture

Standardized frozen hMSCs from multiple donors were obtained from the Tulane Center for Gene Therapy and cultured as previously described^71,72^. The hMSCs were isolated from the bone marrow of healthy donors ranging in age from 19 to 49 years based on plastic adherence, negative for CD34, CD45, CD117 (all less than 2%) and positive for CD29, CD44, CD49c, CD90, CD105, and CD147 markers (all greater than 95%), and possess tri-lineage differentiation potential upon induction *in vitro*^73,74^. Briefly, hMSCs were expanded at a density of 1.7 × 10^3^ cells/cm^2^ using αMEM (Invitrogen) medium supplemented with 10% fetal bovine serum (FBS) and 1% penicillin/streptomycin. At approximately 80% confluence, adherent cells were harvested with 0.25% trypsin-EDTA (Sigma-Aldrich) and further propagated.

### Endothelial differentiation and neural differentiation from hiPSCs in suspension

#### Generation of endothelial cell (iEC) spheroids

Undifferentiated iPSK3 cells were seeded in U-bottom ultra-low-attachment (ULA) 96-well plates (Corning Inc.) at 1 × 10^4^ per well (unless otherwise noted) in differentiation medium composed of RPMI plus 2% B27 (Life Technologies) for 3 days. Y27632 (10 µM) was added during the seeding and removed after 24 h. Then Wnt activator CHIR99021 (10 μM, StemCell Technologies Inc.) was added to the culture medium for 5 days followed by another 6 days in medium without CHIR99021^75–77^. iEC aggregates (day 14) were characterized or transferred to the wells containing iNPC spheroids. The iEC spheroids expressed endothelial markers CD31 and VE-cadherin (Supplementary Fig. S1).

#### Generation of cortical iNPC spheroids

Undifferentiated iPSK3 cells (0.5–2 × 10^4^ cells) were seeded into U-bottom ULA 96-well plates in neural differentiation medium composed of DMEM/F-12 plus 2% B27 in the presence of Y27632 (10 µM). The aggregates were treated with 10 µM SB431542 (Sigma) and 100 nM LDN193189 (Sigma) for 7 days^31^. At day 7, the spheroids were treated with retinoic acid (RA) (2 μM, Sigma) and FGF2 (25 ng/mL, Life Technologies) and grown in neural medium for another 7 days^33^. For maturation, cortical spheroids were maintained in neural differentiation medium without growth factors for additional 7–38 days. The NPC spheroids expressed various neural markers including Nestin, TBR1 and β-tubulin III (Supplementary Fig. S2)^33^.

### Hybrid iNPC, iEC and hMSC spheroid formation and neural differentiation

Two different methods with different sequences of adding iEC spheroids or hMSCs to the wells containing iNPC spheroids were evaluated.

#### Tri-culture of iNPC, iEC and hMSC hybrid spheroids using Method A (iNPC-MSC-iEC)

Human MSCs were pre-labeled with CellTracker Red (2.5 μM, Life Technologies) for 30 min unless otherwise noted. Different numbers of hMSCs were added to day 7 iNPC spheroids (with different seeding densities) for a total of 2 × 10^4^ cells (Fig. 1A) or directly mixed at day 0^30^. The ratios of hiPSC: hMSCs were 4:0, 3:1, 2:2, and 1:3 based on initial seeded cell numbers. Then, the day 14 iEC spheroids were transferred into the wells containing day 14 iNPC-MSC spheroids. The ratios of difference cell types were iNPC: iEC: MSC = 4:2:0, 3:2:1 2:2:2, and 1:2:3 (total 4 × 10^4^ per well, n = 3–12), **referred as Bi-(4:2), Tri-(3:2:1), Tri-(2:2:2), and Tri-(1:2:3)** respectively. For some experiments, iNPC only^32,33^, iEC only^16,78^ or MSC only spheroids^28,48,79^ were used as controls. The hybrid iNPC-MSC-iEC spheroids were maintained in neural differentiation medium for additional 7 days for aggregate fusion.

#### Tri-culture of iNPC, iEC and hMSC hybrid spheroids using Method B (iNPC-iEC-MSC)

For this sequence, day 14 iEC spheroids were transferred into the wells containing day 14 iNPC spheroids (Fig. 1A). Immediately after the transfer, hMSCs were added into the wells at different cell densities to maintain ratios of i**NPC: iEC: MSC = 4:2:0, 3:2:1, 2:2:2, and 1:2:3** (n = 3–12). For long-term cultures, day 14 iEC spheroids were transferred into the wells containing day 14 iNPC spheroids to allow aggregate fusion. Then, at 7 days before harvesting, hMSCs were added into the wells at different cell densities to maintain ratios of iNPC: iEC: MSC = 4:2:0, 3:2:1 2:2:2, 1:2:3, and 0:2:4. The total culture length ranged from 21 days to 52 days.

### Effects of Geltrex, hyaluronic acid (HA) hydrogels, and ROCKi Y27632 on spheroid fusion

To form HA hydrogels, 1% (w/v) HA (Sigma) solution was reacted with 5-fold molar excess amount of methacrylic anhydride (sigma) for 15 h in the dark at 4 °C. The final product was collected by precipitating the solution in 5-fold volume of ethanol twice and purified by dialysis using a membrane (3.5 kDa Mw cut-off, Thermofisher) to remove unreacted reagents. Purified MA-HA was filtered, lyophilized, and stored at −20 °C until further use^80^. For culture experiments, day 14 iEC spheroids were transferred to the wells containing day 14 iNPC spheroids, at the same time hMSCs were added into the wells at different ratios (iNPC: iEC: MSC = 4:2:0, 3:2:1 2:2:2, and 1:2:3). The aggregates were cultured in medium with different concentrations of GelTrex (5%, or 10% v/v) or HA hydrogel (0.025 or 0.05 wt %) or treated with ROCK inhibitor Y-27632 (20 or 40 μM) at day 0. The morphology of hybrid spheroids were captured over 7 days by a phase contrast microscope.

### Aggregate fusion analysis

The images of spheroid fusion were captured over time by a phase contrast microscope. The captured images were converted to binary images using ImageJ software (http://rsb.info.nih.gov/ij) and analyzed with the “particle analysis tool”. Through particle analysis, the squared aspect ratio of contact length between the two aggregates over maximum diameter (Fig. 1C(i)) was calculated to indicate the aggregate fusion process. The inter-sphere angle was measured as the intersecting angle between the tangent lines of two contacted spheroids to the touch point. At least three images were analyzed for each data point. For some experiments, the number of branching points and the total tube length in the images were evaluated.

### Biochemical assays

#### MTT assay

The spheroids of different conditions were incubated with 5 mg/mL 3-(4,5-Dimethylthiazol-2-yl)-2,5-diphenyltetrazolium bromide (MTT, Sigma) solution at day 7 after tri-culture unless otherwise noted. The absorbance of the samples was measured at 500 nm using a microplate reader (Biorad, Richmond, CA).

#### DNA assay

The DNA content of the hybrid spheroids was determined at day 7 after three cell types were co-cultured unless otherwise noted. DNA standard was prepared by dissolving salmon testes DNA in TEX (10 mM Tris, 1 mM EDTA, 0.1% Triton X-100 at pH 8) and a standard curve was constructed for each assay. The aggregates were lysed with 0.1 mg/mL proteinase K (Fisher Scientific, Pittsburgh, PA) at 50 °C overnight. The lysates (100 μL) were mixed with 100 μL of Picogreen (Molecular Probes) in a 96-well plate. The plate was incubated for 5 min in the dark and then read on a fluorescent plate reader (FLX800, Bioinstrument Inc., Winooski, VT).

#### Enzyme-linked immunosorbent assay (ELISA) assay

To quantify the growth factors secreted by different spheroids, culture supernatants were collected at day 7 after three cell types were co-cultured. Concentrations of FGF2, PGE2, VEGF, and TGF-β1 were measured by ELISA according to the manufacturers’ instructions (R&D Systems, Minneapolis, MN for PGE2 and FGF2; Life Technologies for TGF-β1 and VEGF).

### Immunocytochemistry

Briefly, the samples were fixed with 4% paraformaldehyde (PFA) and permeabilized with 0.2–0.5% Triton X-100. The samples were then blocked for 30 min and incubated with various mouse or rabbit primary antibodies (Supplementary Table S1) for four hours. For surface markers, no permeabilization was performed. After washing, the cells were incubated with the corresponding secondary antibody: Alexa Fluor® 488 goat anti-Mouse IgG_1_, Alexa Fluor® 488 or 594 goat anti-Rabbit IgG, or 594 donkey anti-goat IgG (Life Technologies) for one hour. The samples were counterstained with Hoechst 33342 and visualized using a fluorescent microscope (Olympus IX70, Melville, NY) or a confocal microscope (Zeiss LSM 880).

### Histology

For histology, various (about 5–10) spheroids/organoids (day 21 or day 47) were fixed in 10% formalin, dehydrated, and embedded in paraffin wax. The sections of 10 μm were cut and stained with Lerner-2 Hematoxylin (Lerner Laboratories, Pittsburgh, PA) and Eosin-Y w/Phloxine (Richard-Allan Scientific, Kalamazoo, MI)^48^. The sections were also stained with anti-CD31, β-tubulin III, zona occludens 1 (ZO1), HOXB4, TBR1, SATB2, BRN2, Collagen IV and laminin (Supplementary Table S1) to show cellular distribution and cortical layer formation. Images were captured with an Olympus IX70 microscope or a confocal microscope (Zeiss LSM 880).

### Flow cytometry

To quantify the levels of various markers, the cells were harvested by trypsinization (0.05% trypsin for 10–15 min followed by pipetting with micro-tips) and analyzed by flow cytometry^81^. Briefly, 1 × 10^6^ cells per sample were fixed with 4% PFA and washed with staining buffer (2% FBS in PBS). The cells were permeabilized with 100% cold methanol, blocked, and then incubated with primary antibodies against β-tubulin III, KDR, CD31, and VE-cadherin, followed by the corresponding secondary antibody Alexa Fluor 488 goat anti-Mouse IgG_1_ (for β-tubulin III, KDR), or Alexa Fluor 594 donkey anti-goat IgG (for CD31, VE-Cadherin^82^). For surface markers, no permeabilization was performed. The cells were acquired with BD FACSCanto™ II flow cytometer (Becton Dickinson) and analyzed against isotype controls using FlowJo software.

### Reverse transcription polymerase chain reaction (RT-PCR) analysis

Total RNA was isolated using the RNeasy Mini Kit (Qiagen, Valencia, CA) according to the manufacturer’s protocol followed by the treatment of DNA-Free RNA Kit (Zymo, Irvine, CA)^83^. Reverse transcription was carried out using 2 μg of total RNA, anchored oligo-dT primers (Operon, Huntsville, AL), and Superscript III (Invitrogen, Carlsbad, CA) (according to the protocol of the manufacturer). Primers specific for target genes (Supplementary Table S2) were designed using the software Oligo Explorer 1.2 (Genelink, Hawthorne, NY). The gene β-actin was used as an endogenous control for normalization of expression levels. Real-time RT-PCR reactions were performed on an ABI7500 instrument (Applied Biosystems, Foster City, CA), using SYBR1 Green PCR Master Mix (Applied Biosystems). The amplification reactions were performed as follows: 2 min at 50 °C, 10 min at 95 °C, and 40 cycles of 95 °C for 15 sec and 55 °C for 30 sec, and 68 °C for 30 sec. Fold variation in gene expression was quantified by means of the comparative Ct method: ${2}^{-({C}_{ttreatment}-{C}_{tcontrol})}$, which is based on the comparison of expression of the target gene (normalized to the endogenous control β-actin) between the hybrid spheroids and the spheroids of NPC only.

### Effect of AMD3100

The Day 14 hiPSC-EC spheroids were labeled with CellTracker Red. The hMSCs were labeled with CellTracker Green. The hybrid (iNPC-iEC-MSC) spheroids were cultured in neural differentiation media (control) or media containing the CXCR4 inhibitor AMD3100 (100 nM, Sigma) for additional 10 days^84^. The fusion kinetics and cell localization were captured over time. The cell viability of day 10 hybrid spheroids was determined by MTT activity assay.

### Whole-patch clamping for electrophysiology

Whole-cell patch clamp was used to record from iPSK3-derived spheroids cultured on glass covered slips. Cover slips were washed three times with extracellular recording solution containing (in mM) 136 NaCl, 4 KCl, 2 MgCl, 10 HEPES, and 1 EGTA (312 mOsm, pH 7.39) and were incubated in this solution at room temperature during recording. Glass electrodes (resistance 1–5 MΩ) were filled with intracellular solution containing 130 mM KCl, 10 mM HEPES, and 5 mM EGTA (292 mOsm, pH 7.20). Cells were visualized under phase contrast with a Nikon Eclipse Ti-U inverted microscope and attached DS-Qi1 monochrome digital camera. Recordings were made with an Axopatch 200B amplifier (Molecular Devices) and digitized with a Digidata 1440 A system (Molecular Devices). Ionic currents were recorded under a voltage clamp protocol (−60 mV to 135 mV in 15 mV steps, 250 ms in duration). Action potentials were recorded under a current clamp protocol (−100 pA to 200 pA in 20 pA steps, 800 ms in duration). Spontaneous post-synaptic currents were recorded under continuous voltage clamp at −80 mV for 2 min. Signals were filtered at 1 kHz and sampled at 10 kHz. Data was collected and analyzed using pCLAMP 10 software (Molecular Devices).

### Statistical analysis

Each experiment was carried out at least three times (using different batches of cells) with triplicate samples (in some cases spheroids were pooled from more than 12 wells) in each experiment. The representative experiments were presented and the results were expressed as [mean ± standard deviation]. To assess the statistical significance, one-way ANOVA followed by Fisher’s LSD post hoc tests were performed. A *p*-value < 0.05 was considered statistically significant.

## Supplementary information

Supplementary Materials unmarked
