# Supplementary material for: Assembly of Human Stem Cell-Derived Cortical Spheroids and Vascular Spheroids to Model 3-D Brain-like Tissues
Source: Sci Rep. 2019 Apr 12;9:5977. doi: 10.1038/s41598-019-42439-9 (PMC6461701; doi:10.1038/s41598-019-42439-9)
Supplement: Supplementary file 1 — Supplementary Materials unmarked [file 41598_2019_42439_MOESM1_ESM.docx]

**Supplementary Materials**

**Assembly of Human Stem Cell-Derived Cortical Spheroids and Vascular Spheroids to Model 3-D Brain-like Tissues**

Liqing Song^1^, Xuegang Yuan^1^, Zachary Jones^2^, Kyle Griffin^1^, Yi Zhou^2^,

Teng Ma^1,^ *, Yan Li^1,^ *

^1^Department of Chemical and Biomedical Engineering; FAMU-FSU College of Engineering; Florida State University; Tallahassee, FL USA

^2^Department of Biomedical Sciences, College of Medicine, Florida State University, Tallahassee, Florida, USA

**Supplementary Information 1.**

***Generation of iNPC-iEC spheroids*:** The day 12-14 iEC spheroids were transferred into the wells containing day 12-14 iNPC spheroids in ultralow-attachment (ULA) 96-well plates. The hybrid spheroids were maintained in neural medium for another 7 days and the fusion process was captured by phase contrast microscope over the last 7 days.

**Results:** The fusion of iEC spheroids and iNPC spheroids was characterized firstly. Cell Tracker Red labeled iEC spheroids and the cortical iNPC spheroids merged into a spherical aggregate with a squared aspect ratio of 0.7~0.9 by day 5 (**Supplementary Figure S3**). The iNPC-iEC hybrid spheroids showed density-dependent MTT activities. Next, hMSCs (labeled with CellTracker Red) were included in the co-culture system. Two methods were evaluated: (A) iNPC-MSC-iEC; and (B) iNPC-iEC-MSC. To construct iNPC-MSC-iEC hybrid spheroids, hMSCs were added to day 7 preformed iNPC spheroids and grown for another 7 days (**Supplementary Figure S4A**) or hiPSCs and hMSCs were directly mixed at day 0 (**Supplementary Figure S4B**) [1].

**Supplementary Figure S1. Endothelial differentiation from hiPSCs in suspension to generate iEC spheroids.** (A) Schematic illustration of endothelial differentiation protocol. (B) Vascular markers, CD31 and VE-cadherin (VECAD), expression was quantified by flow cytometry. Black line: negative control; red line or blue line: marker of interest from two differentiations. (C) Day 11 vascular spheroids were replated for one day and immunocytochemistry was performed for vascular markers, CD31 (red) and VECAD (red). Hoechst 33342: blue. Scale bar (green): 50 μm. Day 14 vascular spheroids were harvested and prepared for histology sections. (D) Haemotoxylin and Eosin (H&E) staining and (E) CD31 staining. Scale bar: 100 μm. (F) Vascular network formation was observed for day 14 iEC spheroids replated on 1:1 GelTrex-coated surface in endothelial medium and cultured for 7 days. Scale bar: 100 μm.

Briefly, 24-well plates were coated with 200 μL/well 1:1 diluted Geltrex for more than 30 min. The cells were plated at 5×10^5^ cells on Geltrex-coated plates in 500 μL EGM-2 medium (Lonza, for endothelial cells) and incubated at 37^o^C in 5% CO_2_ for 7 days. Cell morphology was photographed by a phase-contrast microscope.

(G) (i) VE-cadherin staining of iEC after 30 days of differentiation; Scale bar: 100 μm. (ii) VE-cadherin (VECAD), expression in day 17 iECs was quantified by flow cytometry.


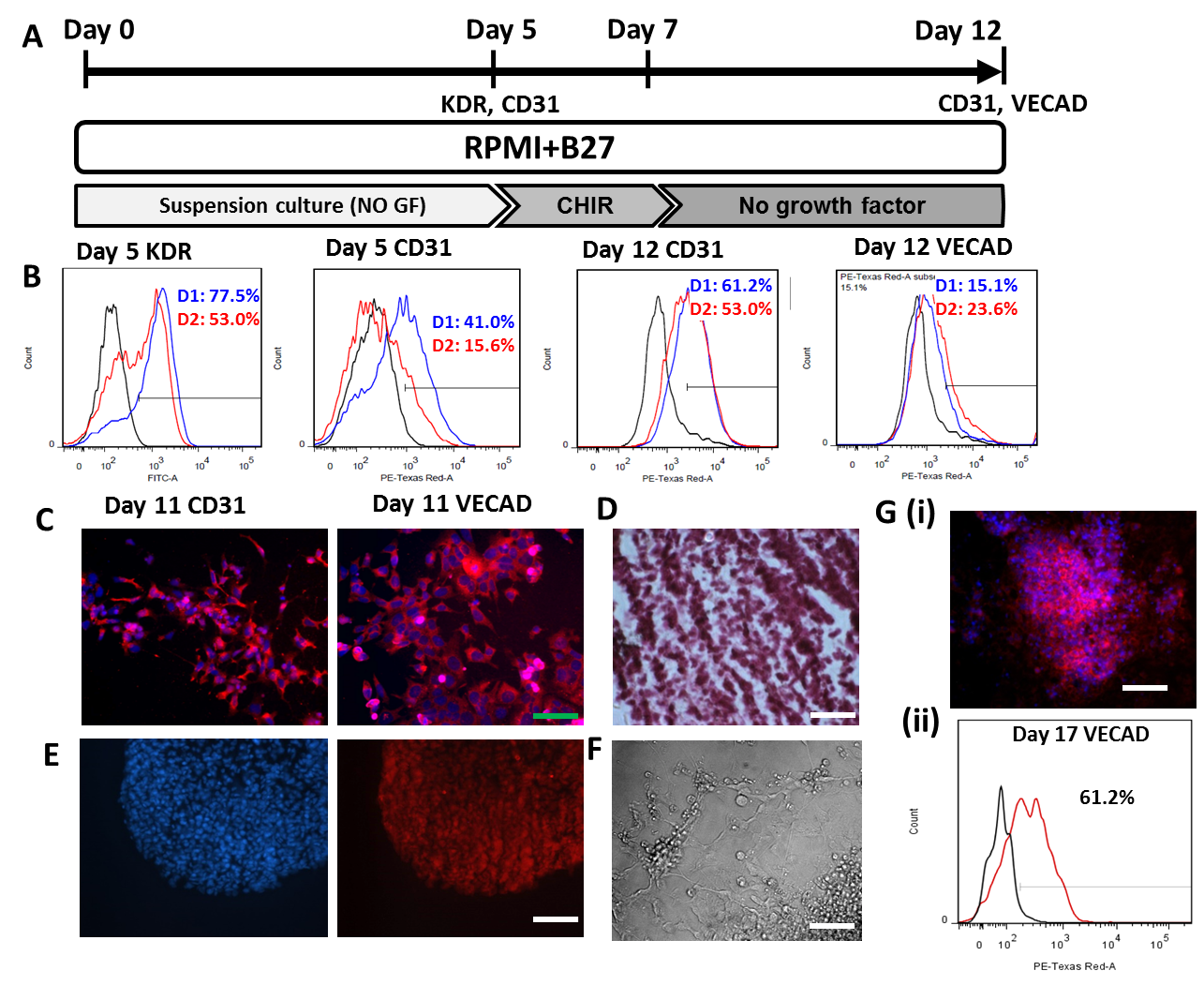


**Supplementary Figure S2. Characterization of neural differentiation from hiPSCs in suspension.** (A) Phase contrast images of iNPC spheroids morphology over 13 days. Scale bar: 200 μm. (B) Confocal images of cortical neuron marker TBR1 and hindbrain marker HOXB4 for day 14 iNPC spheroids. Scale bar: 100 μm. (C) Neural progenitor marker expression Nestin and TBR1 of replated day 14 iNPC spheroids. Scale bar: 100 μm. (D) Neurite outgrowth and axon extension indicated by β-tubulin III expression and phase contrast images of neurons after replating. Scale bar: 100 μm. Different phenotypic marker expression (day 28): (E) Glutamate (red)/Tau (green) and Synapsin-1 (SYN-1) (red)/ β-tubulin III (green), related to neurogenesis. Hoechst 33342: blue. Scale bar: 100 μm. (F) Flow cytometry analysis of Nestin and β-tubulin III (day 20). Black line: negative control; red line: marker of interest.


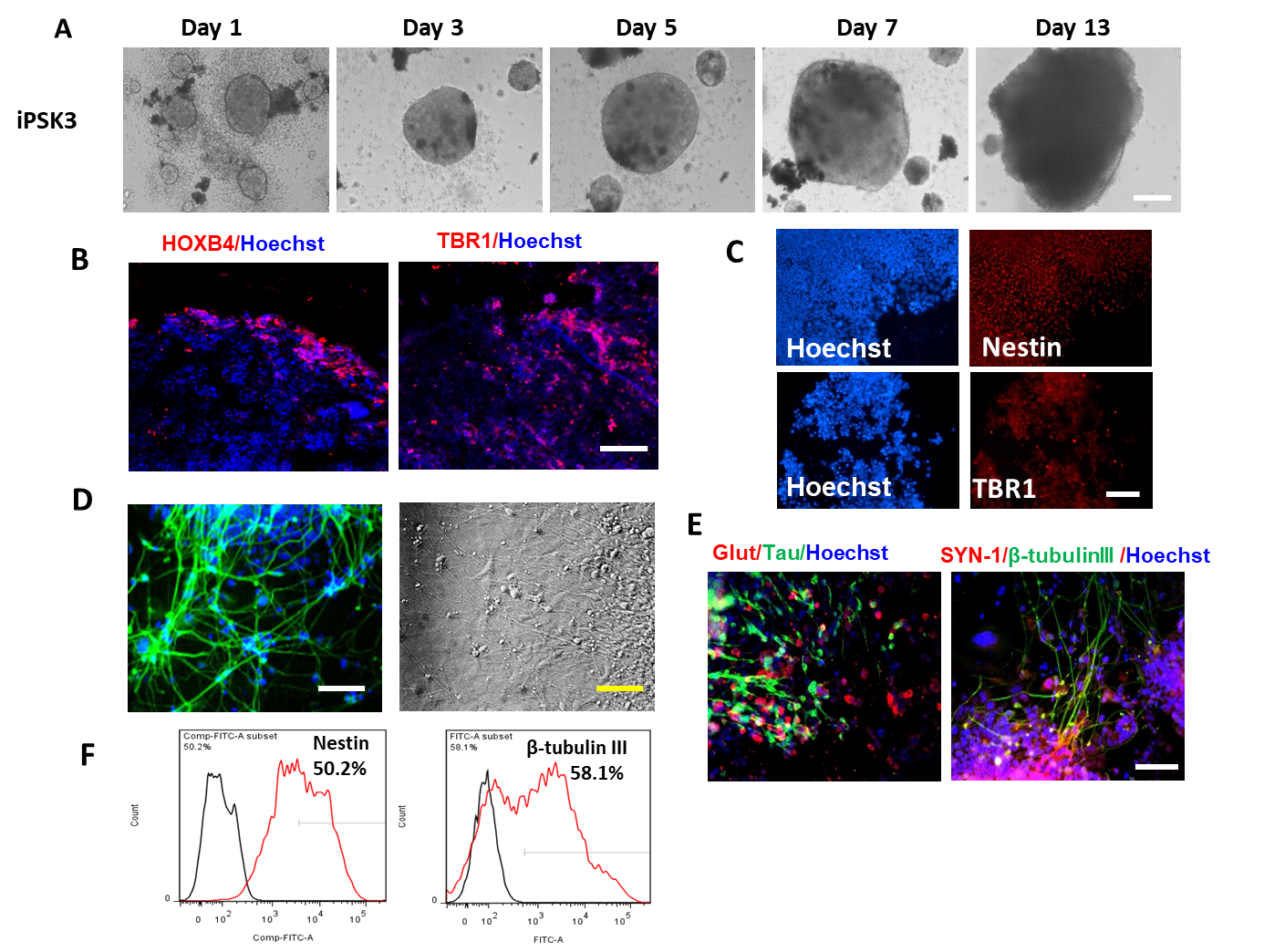


**Supplementary Figure S3. Characterization of neural-vascular spheroids (iNPC-iEC spheroids) derived from hiPSCs in suspension.** (A) Overlay of phase contrast images (iNPCs) with fluorescent images (iECs were labeled with CellTracker Red) of iNPC-iEC spheroids with different seeding densities (cell number per well of 96-wp) at day 11+0, 1, 3, 5. Scale bar: 400 μm. (B) Aggregation kinetics; (C) MTT activities of iNPC-iEC spheroids with different seeding densities. *indicates *p* < 0.05.


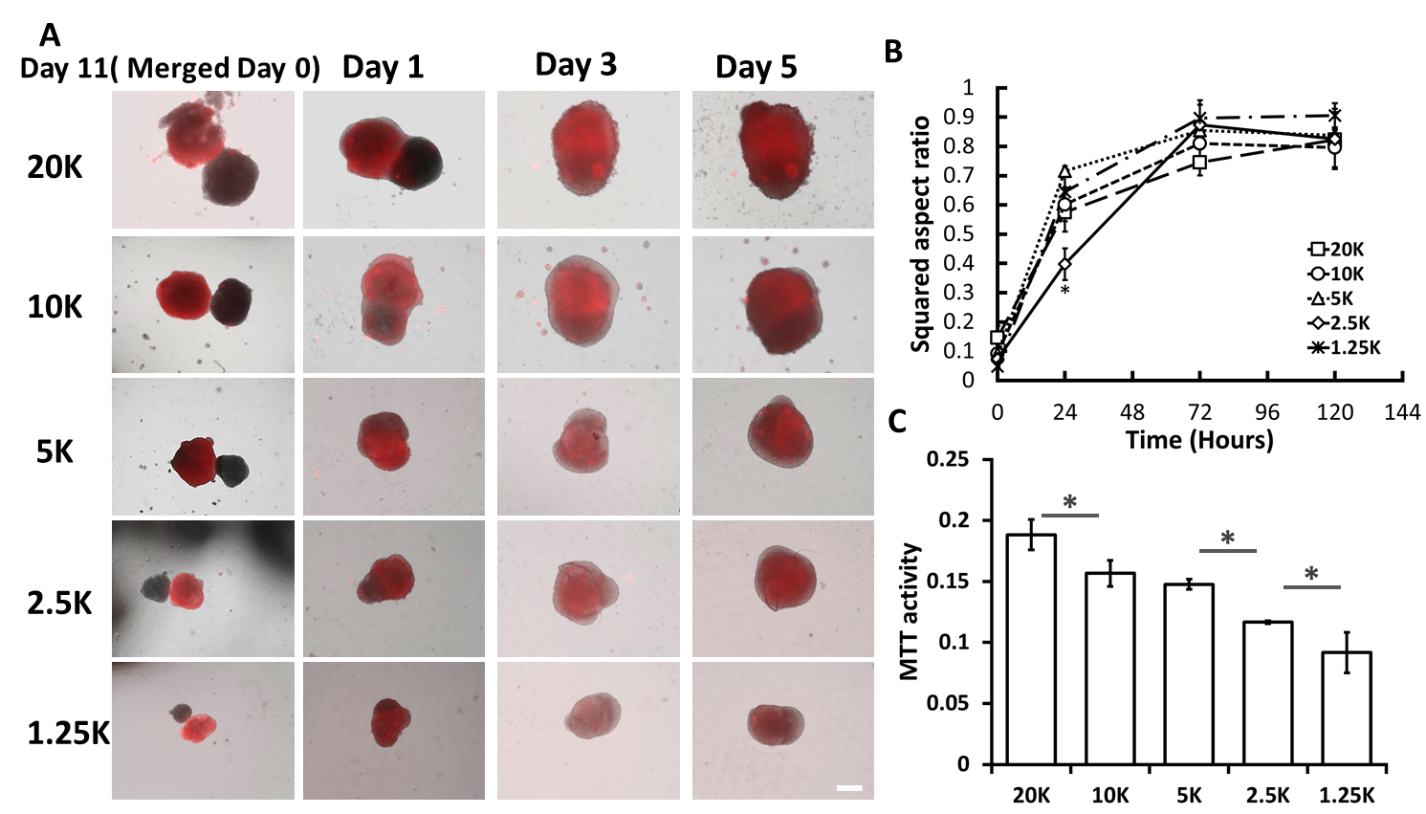


**Supplementary Figure S4A. Effect of different mixing sequence, referred as iNPC-MSC-iEC spheroids, on aggregation kinetics**. hMSCs (labeled with CellTracker Red) were added to day 7 iNPCs for iNPC-MSC-iEC spheroids (method A), before iEC transfer at day 14. (A) Phase contrast images of iNPC-MSC-iEC spheroids morphology at four different cell ratios (total day 14+1, 3, 5, 7 days). (B) Aggregation kinetics of different hybrid spheroids. Scale bar: 400 μm.

**
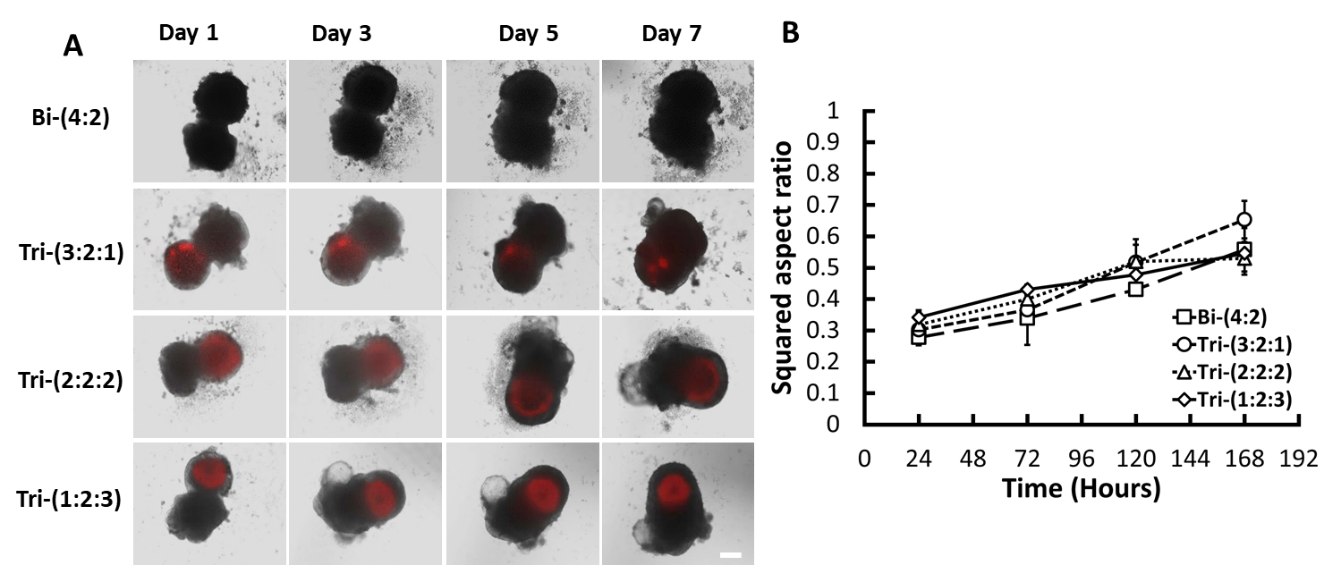
**

**Supplementary Figure S4B. Effect of different mixing sequence, referred as iNPC-MSC-iEC spheroids, on aggregation kinetics.** HiPSCs and hMSCs (labeled with CellTracker Red) were directly mixed at day 0 and cultured for 14 days. Then day 14 iEC spheroids were added to the well. (A) Overlay of phase contrast images (iNPCs and iECs) with fluorescent images (hMSCs labeled with CellTracker Red) of different hybrid spheroids (total day 14+1, 3, 5, 7 days). Scale bar: 400 μm. (B) Aggregation kinetics of different hybrid spheroids. *indicates *p* < 0.05.


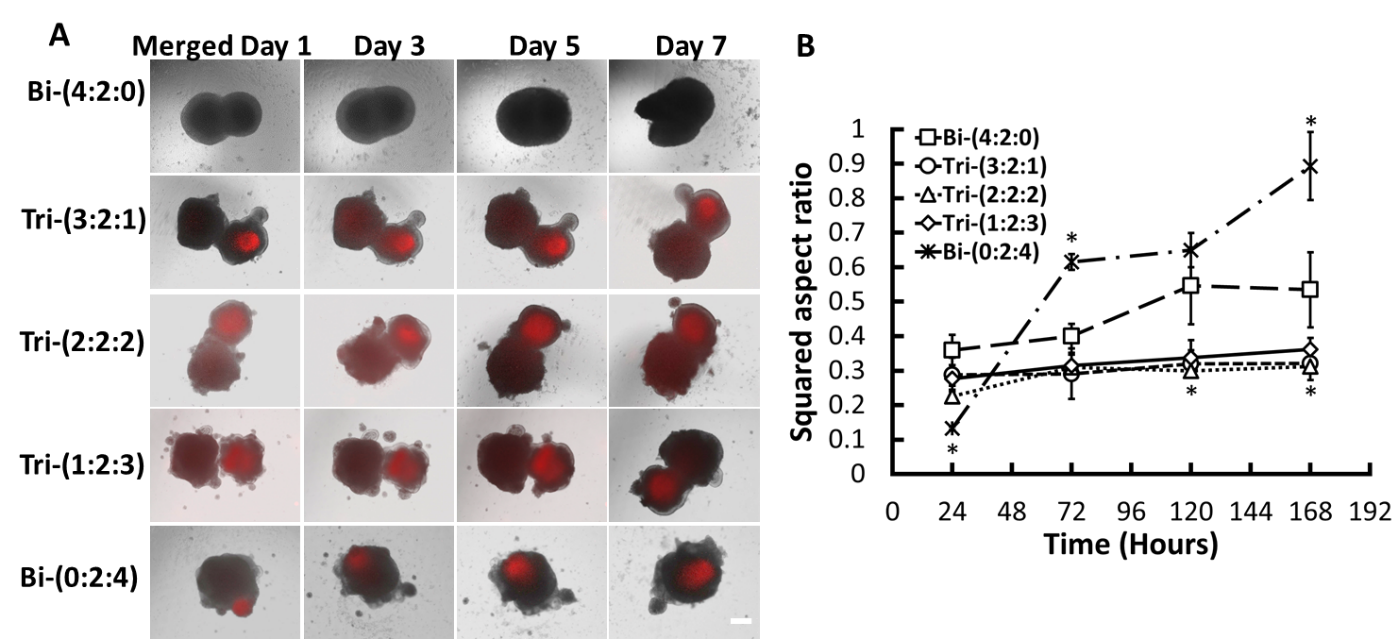


**Supplementary Figure S5. Effects of Geltrex, hyaluronic acid, and ROCKi Y27632 on hybrid spheroid fusion.** hMSCs were labeled with CellTracker Red. (A) (i) Overlay of phase contrast images (iNPC spheroids and iEC spheroids) with fluorescent images (hMSCs) of iNPC-iEC-MSC spheroids with GelTrex at 5% or 10%; (ii) The corresponding aggregation kinetics of hybrid spheroids. (B) (i) Overlay of phase contrast images with fluorescent images of iNPC-iEC-MSC spheroids with hyaluronic acid (HA, 0.025 or 0.05 wt%). (ii) The corresponding aggregation kinetics of hybrid spheroids. (C) (i) Overlay of phase contrast images and fluorescent images of iNPC-iEC-MSC spheroids with the treatment of Y27632 (20 μM or 40 μM). (ii) The corresponding aggregation kinetics of hybrid spheroids. The time when three cell types were co-cultured (day 14) was counted as day 0. Day 1-7 equals total day 15-21. Scale bar: 400 μm. *indicates *p* < 0.05 for the different test conditions.

**
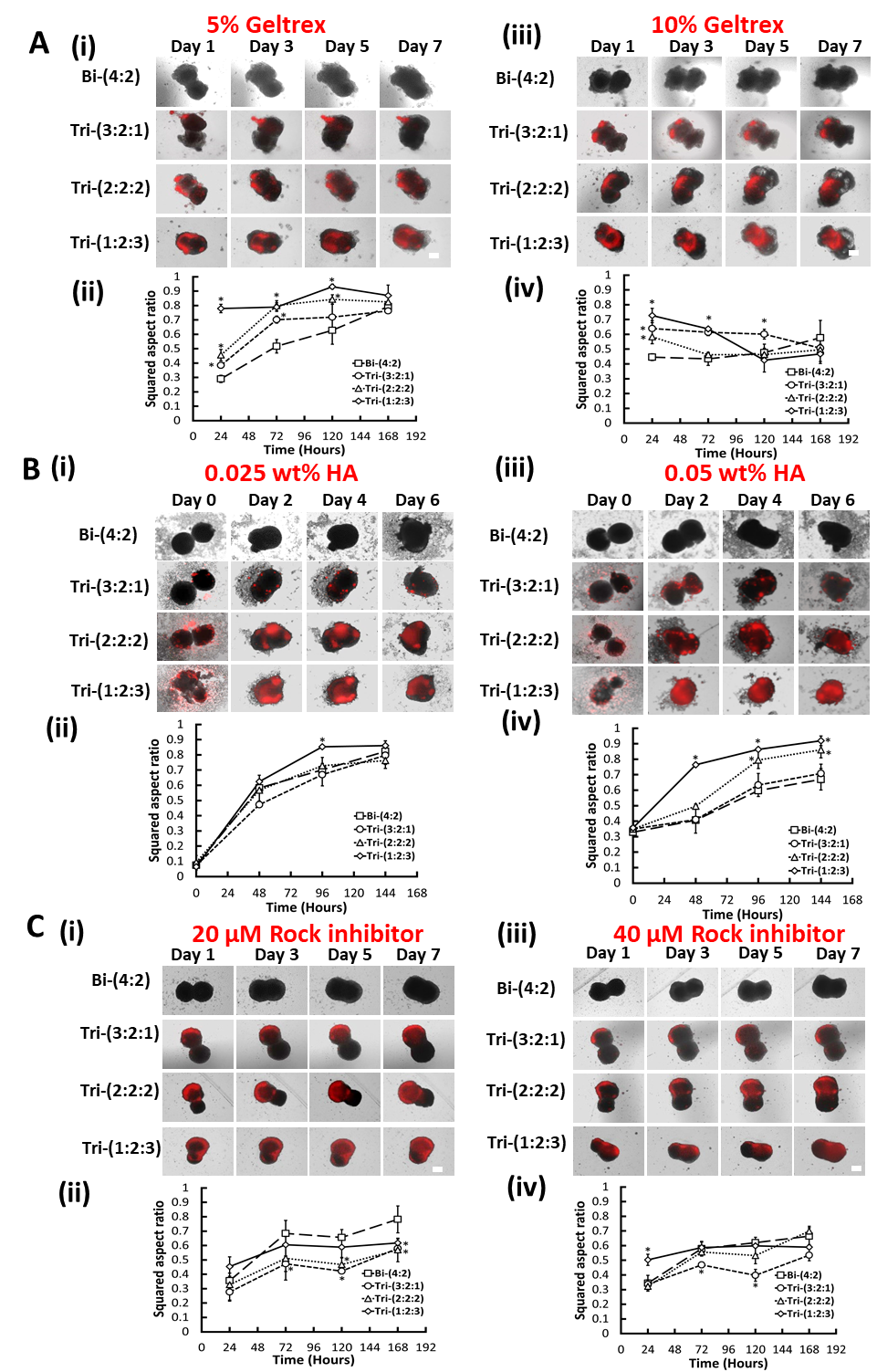
**

**Supplementary Figure S6. Supporting data for hybrid spheroid proliferation.** (A). DNA content of hybrid spheroids with the treatment of 0.025wt% hyaluronic acid (HA) after 7 days (total day 21) of tri-culture compared to control group (no treatment). (B) Confocal images of 5-Bromo-2'-deoxyuridine (BrDU), showing cells in S-phase of cell cycle. Scale bar: 200 μm.

For 5-Bromo-2'-deoxyuridine (BrdU) assay, the cells were incubated in medium containing 10 µM BrdU (Sigma) for four hours. The cells were then fixed with 70% cold ethanol, followed by a denaturation step using 2N HCl/0.5% Triton X-100 for 30 min in the dark. The samples were reduced with 1 mg/mL sodium borohydride for 5 min and incubated with mouse anti-BrdU (1:100, Life Technologies) in blocking buffer (0.5% Tween 20/1% bovine serum albumin in PBS), followed by Alexa Fluor® 488 goat anti-Mouse IgG_1_ (Molecular Probes). The cells were counterstained with Hoechst 33342 and analyzed by a confocal microscope (Zeiss LSM 880).

**
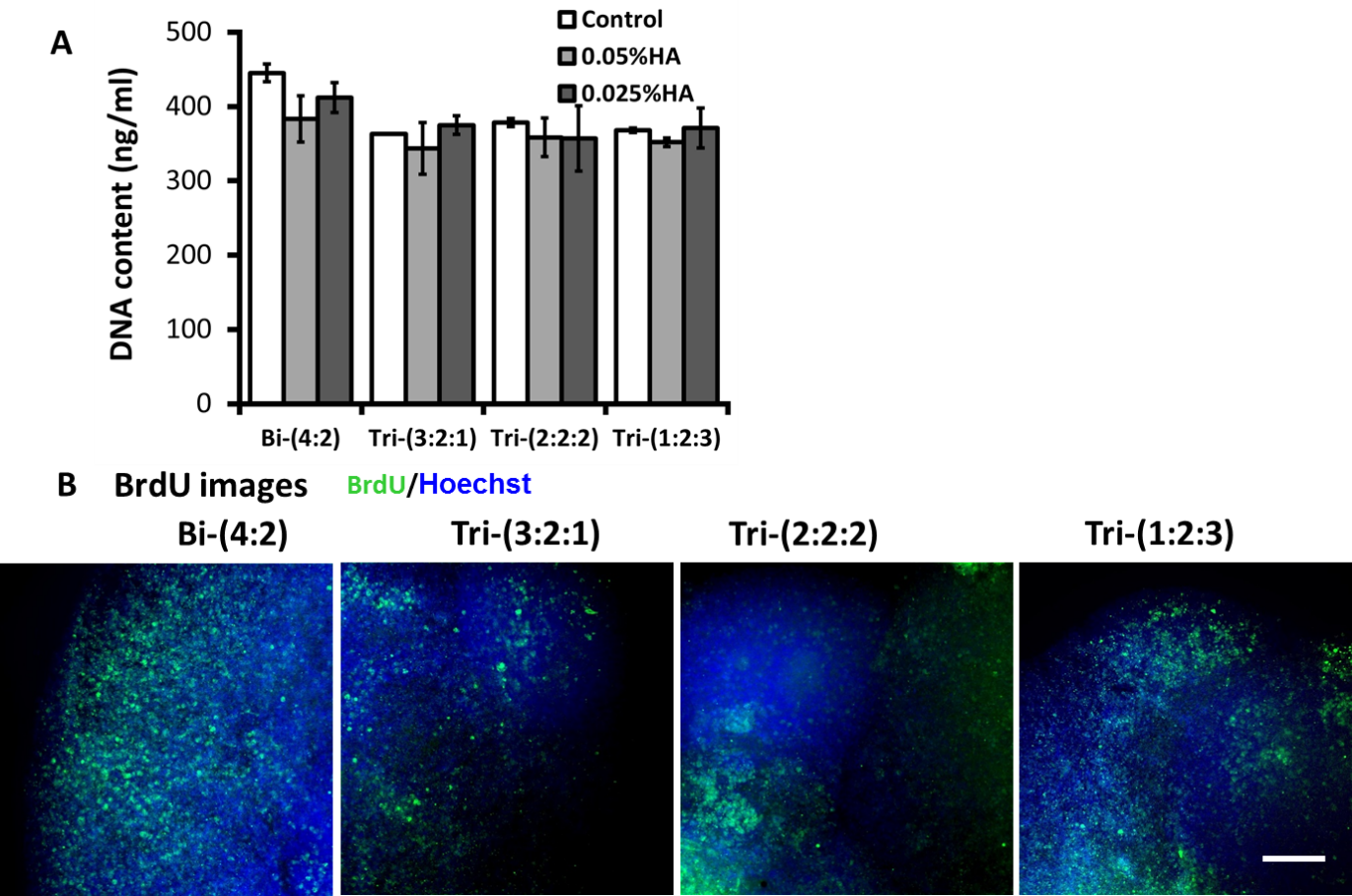
**

**Supplementary Figure S7. VEGF-A secretion of iEC control (supplementary data for Figure 3B).**  iECs secreted much higher VEGF-A ( at ng/ml level) than iNPCs, but much lower than MSC only group in Figure 4B.


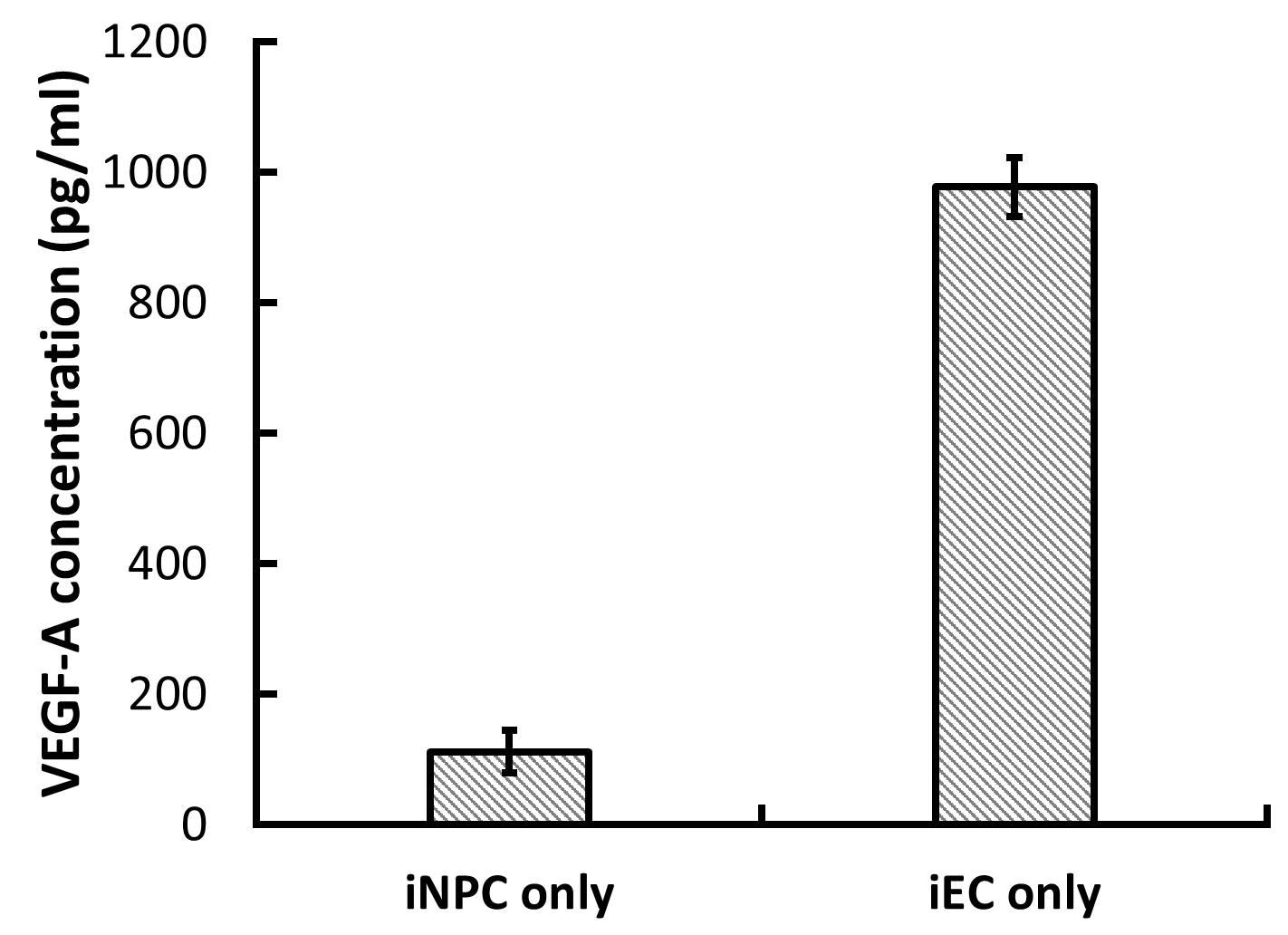


**Supplementary Figure S8. Additional images for Figure 4.** The control images for histology images in Figure 4. (i) BRN2 (red)/ Hoechst (blue) and MAP2 (green)/Hoechst (blue) for iNPC spheroids. (ii) CD31 (red)/Hoechst (blue), VE-cadherin (red)/Hoechst (blue), and ZO1 (green)/Hoechst (blue) for iEC spheroids. Scale bar: 100 μm.


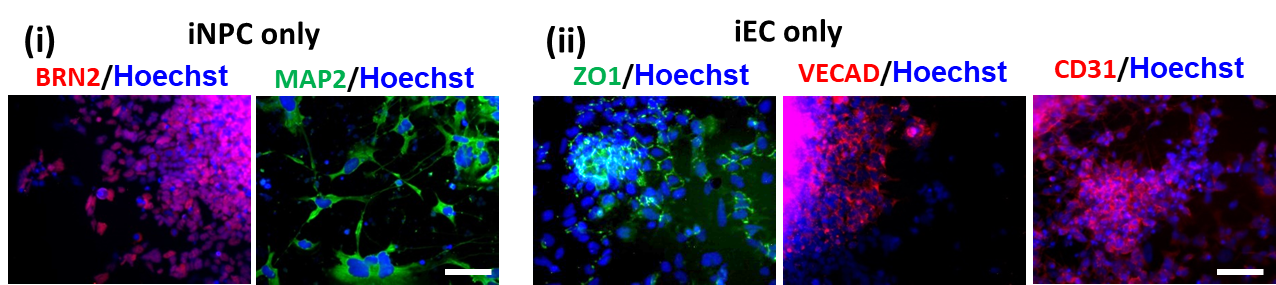


**Supplementary Figure S9. Additional astrocyte marker expression in the spheroids.** Images of histology thin sections of day 47 spheroids for Bi-(4:2) and Tri-(2:2:2) groups: Aldolase C (green)/Hoechst (blue) and vimentin (green)/S100B (red)/Hoechst (blue). Scale bar: 100 μm.

**
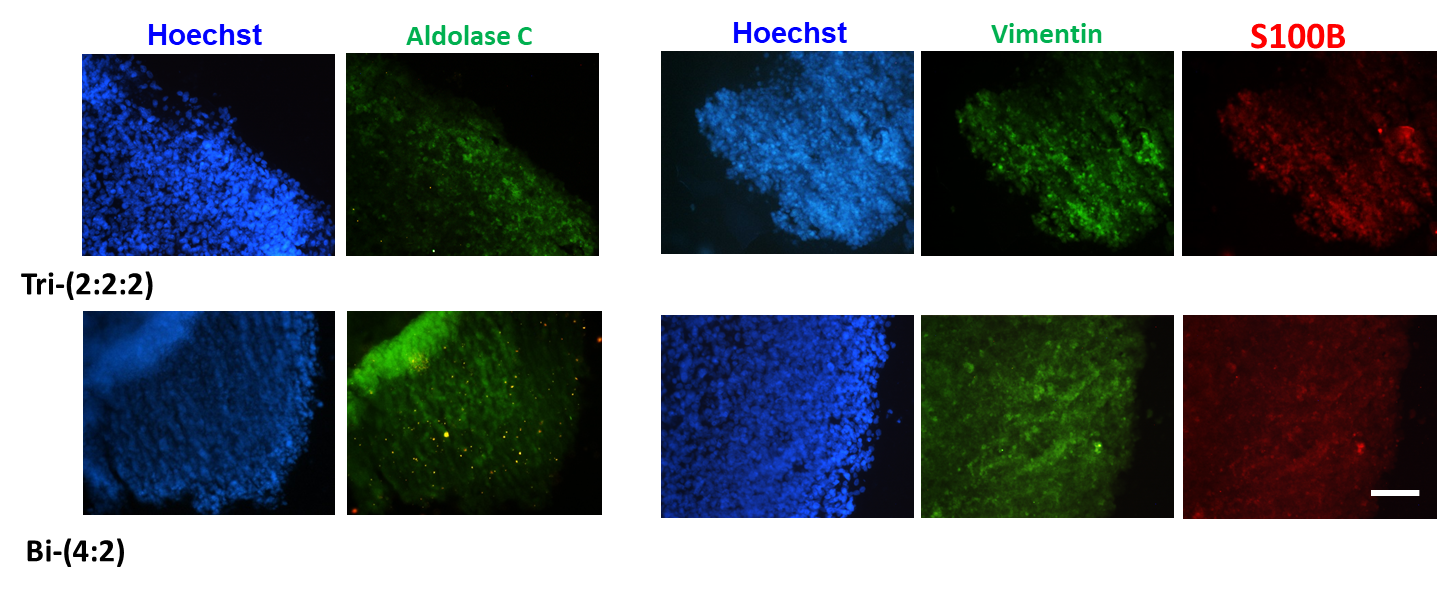
**

**Supplementary Figure S10. Additional images for Figure 5.** Enlarged images for neural marker β-tubulin III (green)/vascular marker CD31 (red) expression in hybrid spheroids of Figure 5A. Scale bar: 100 or 25 μm.


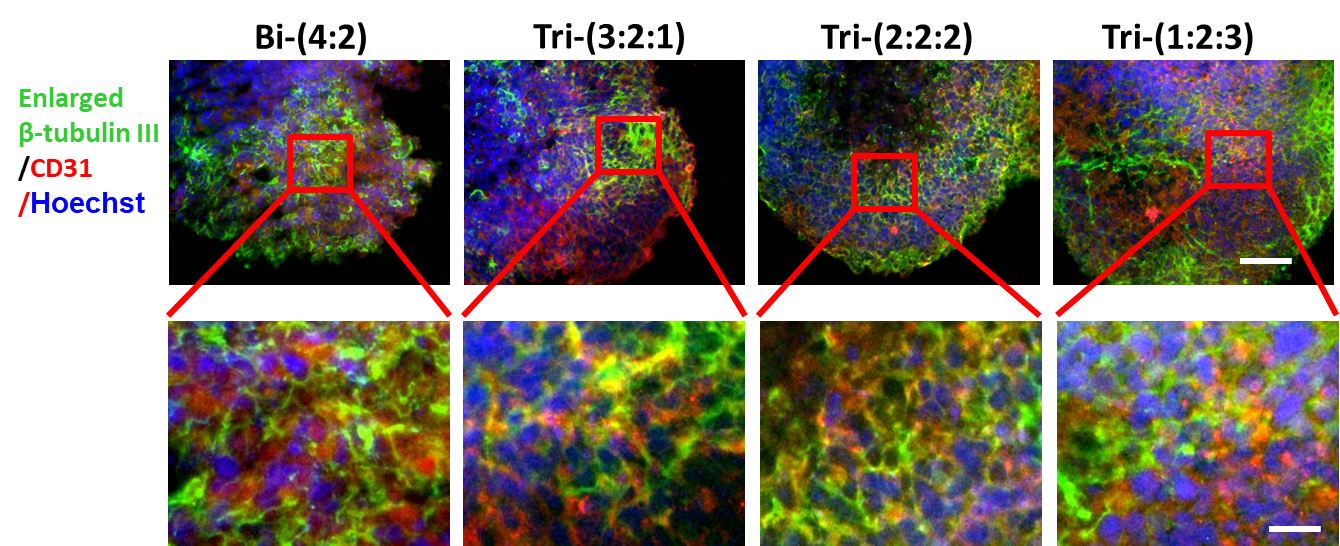


**Supplementary Figure S11. Individual images for Figure 5A.** Scale bar: 100 μm.

**
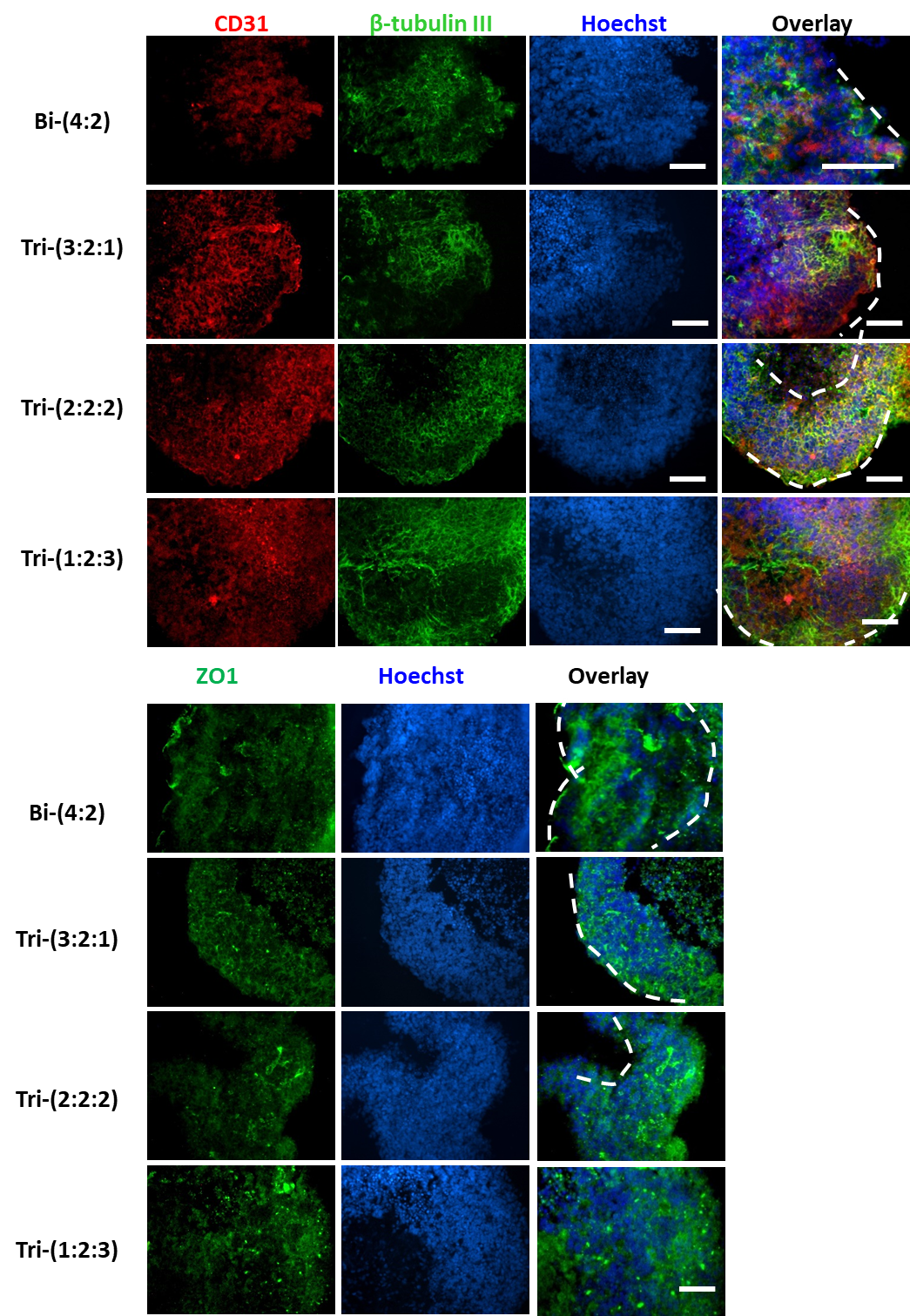
**

**Supplementary Figure S12. Individual images for Figure 5B.** Scale bar: 100 μm.

**
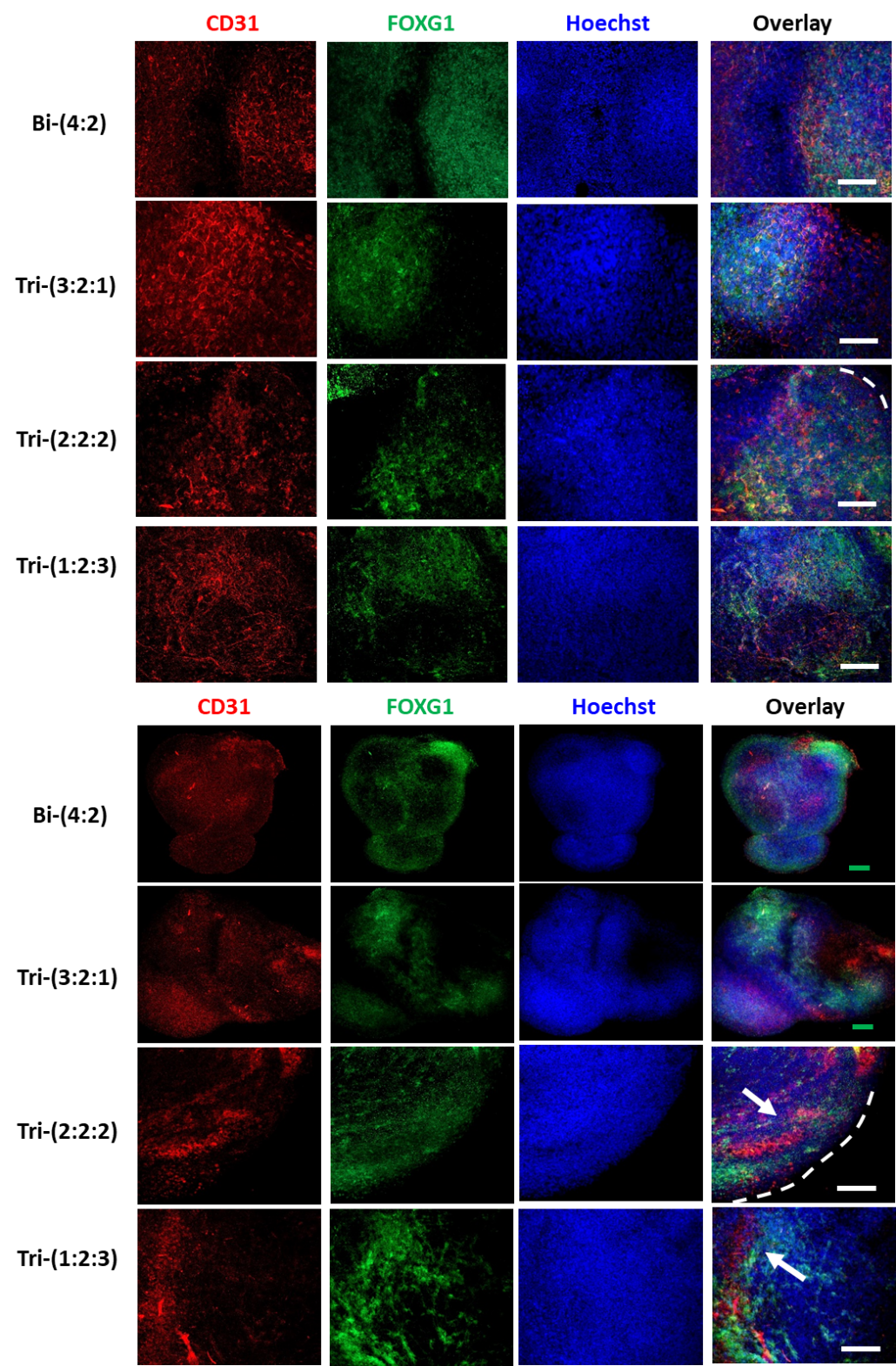
**

**Supplementary Figure S13. Morphology and histology characterizations for early (day 21) and late (day 47) stage hybrid spheroids.** (A) Images of histology thin sections of day 21 spheroids. Cortical layer markers TBR1 (green)/BRN2 (red); HOXB4 (red). Hoechst: blue; Scale bar: 100 μm. (B) Phase contrast images of hybrid spheroid morphology at day 40. Scale bar: 400 μm.


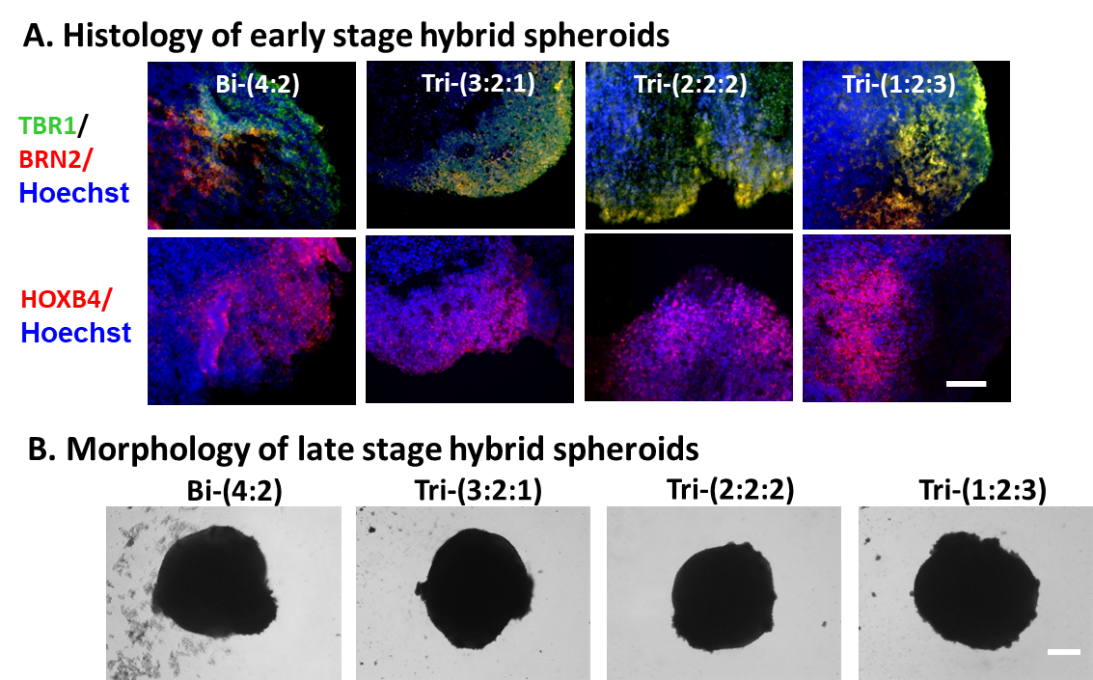


**Supplementary Figure S14. Individual images for Figure 6E.** Scale bar: 100 μm.

**
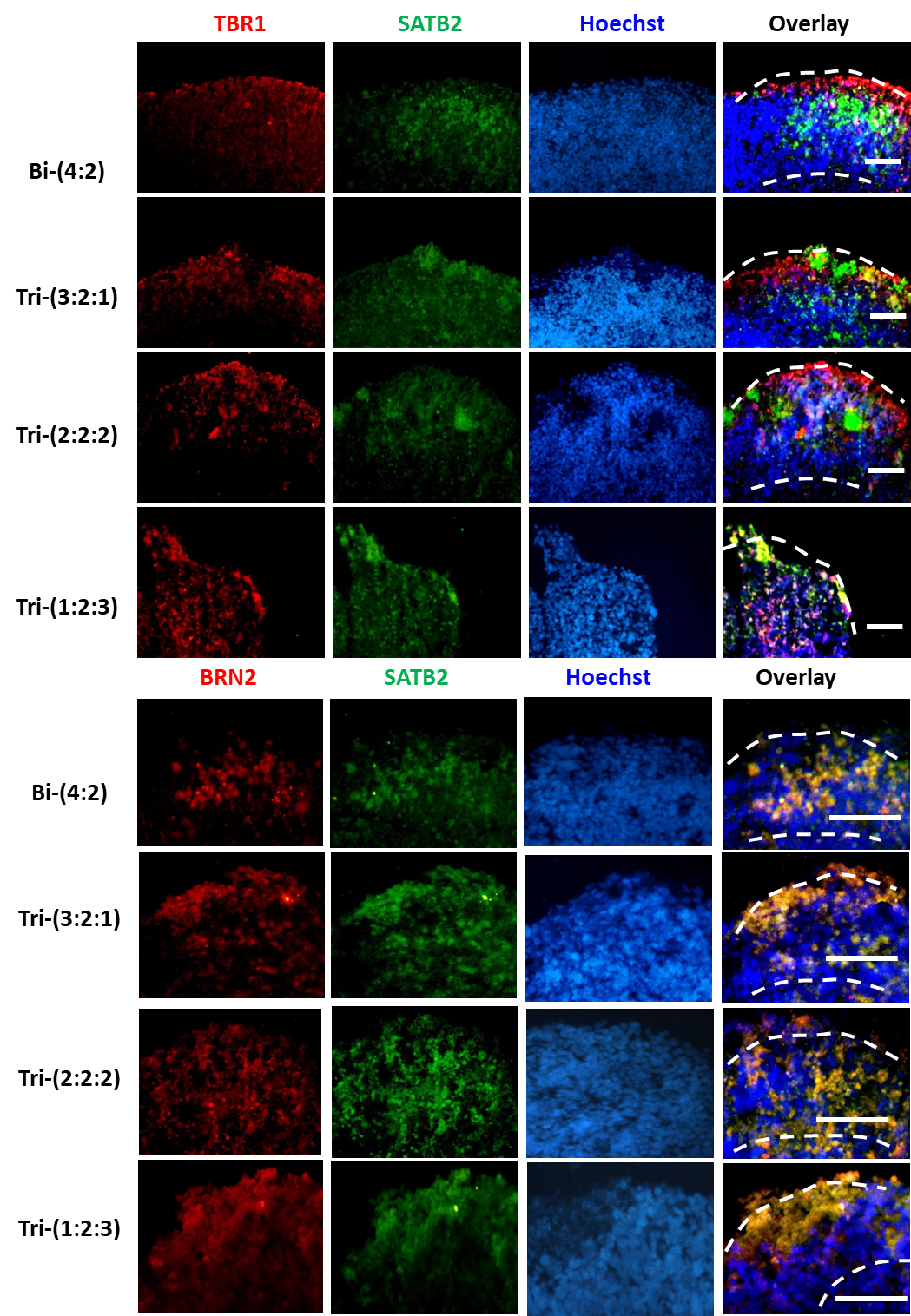
**

**Supplementary Figure S15. Individual images for Figure 6F.** Scale bar: 50 μm.

**
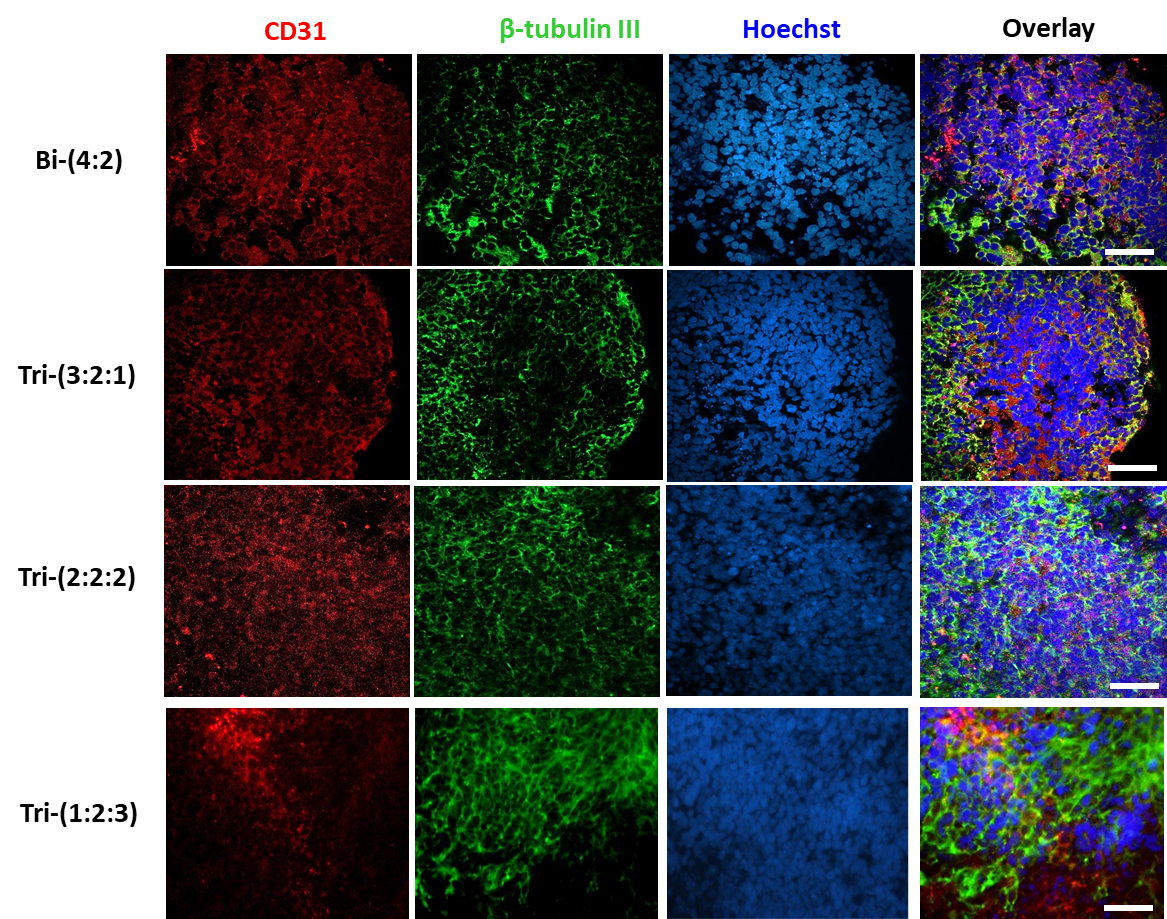
**

**Supplementary Figure S16. Expression of extracellular matrix proteins in late-stage spheroids.** Collagen IV, laminin, chondroitin sulfate proteoglycans (CSPG), hyaluronic acid (HA) expression for hybrid spheroid histological sections at day 47. Scale bar: 100 μm.


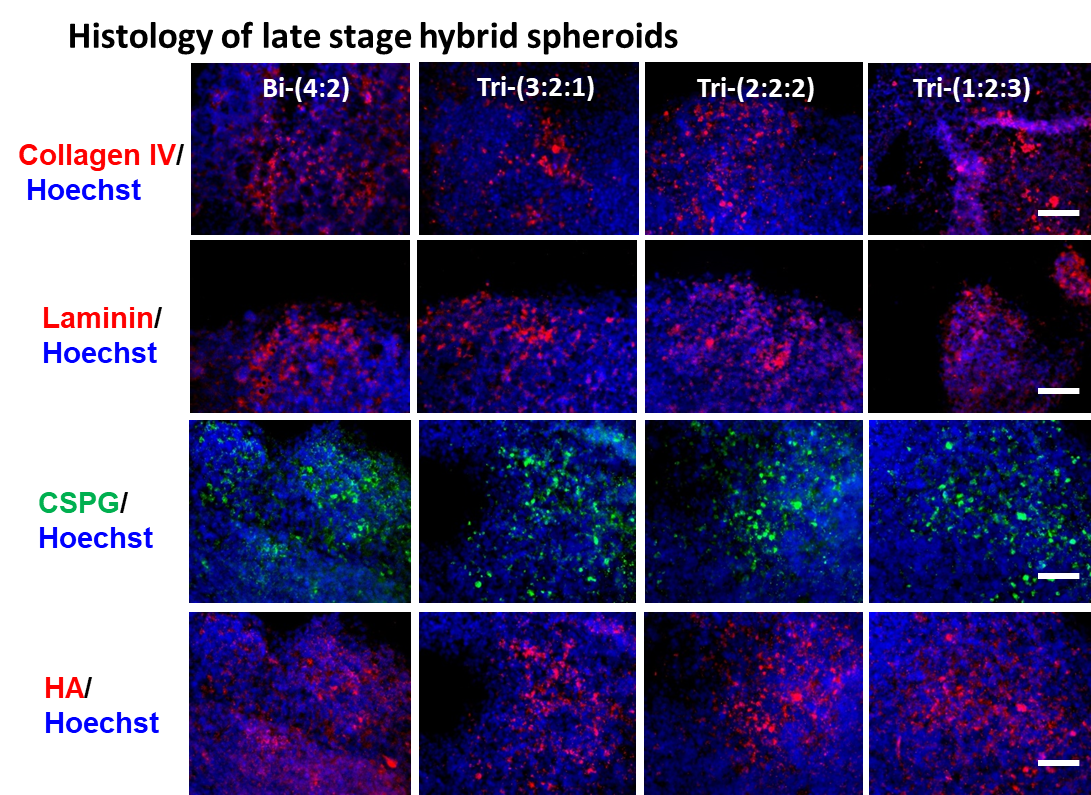


**Supplementary Figure S17. Electrophysiological properties of hybrid spheroids (day 42).** Results were from Bi-(4:2) group. (A) Representative voltage clamp traces showing fast inward Na+ currents and long-lasting outward K+ currents evoked by depolarizing voltage steps. Step size = 15 mV. (B) Representative current clamp traces showing rebound action potentials in response to hyperpolarizing current injections. Step size = 20 pA. (C) Representative trace of continuous voltage clamp recording showing spontaneous postsynaptic currents. (D) Representative phase-contrast images of a recorded neuron outgrowth of the spheroids. Scale bar: 20 μm.


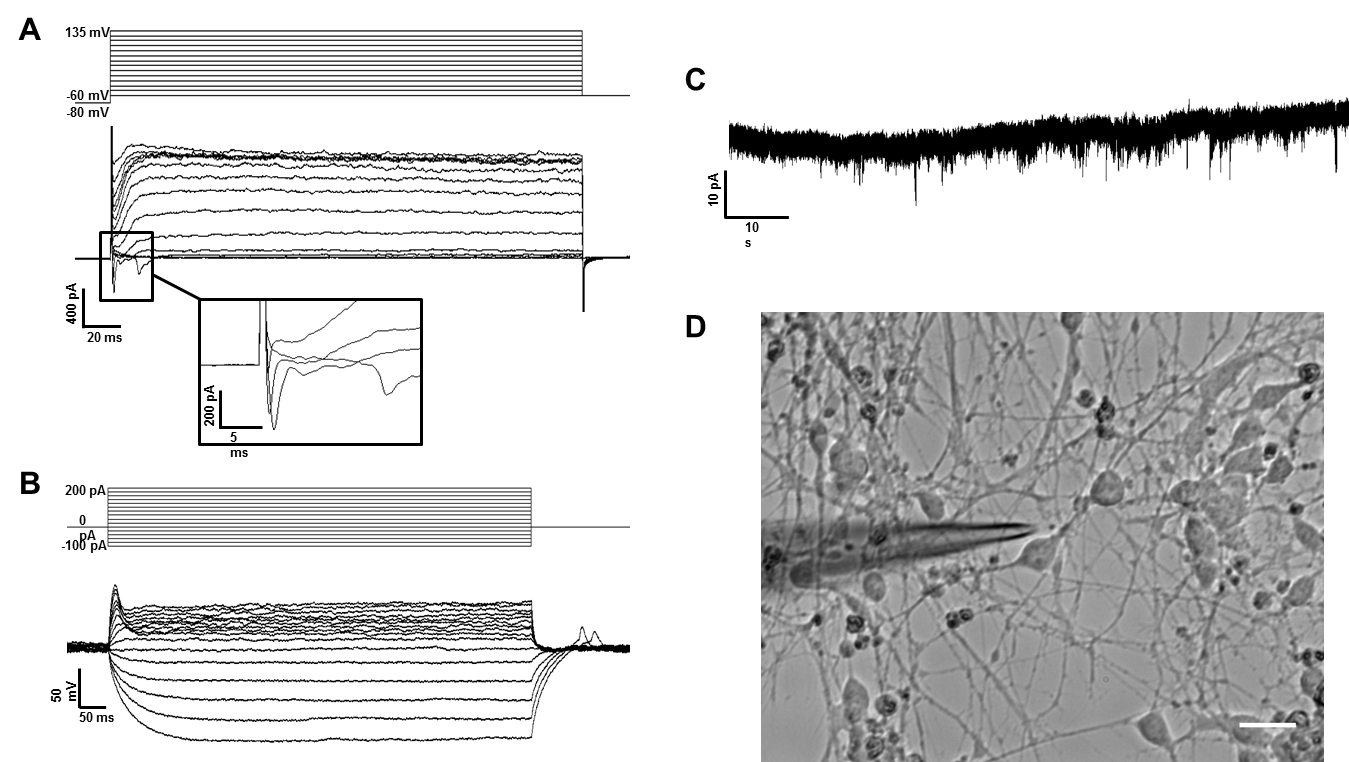


**Supplementary Figure S18. RT-PCR analysis for iEC and hMSC controls compared to iNPC group (supporting data for Figure 7).** (A) Brain regional marker genes; TBR1 and Nkx2.1 were the highest for the iNPC group as expected. For HOXB4, it may not be a specific hindbrain marker. (B) Matrix remodeling and cell-cell communication genes; MMP2 and MMP3 were the highest for the MSC group as expected, then iEC group, and iNPC group. (C) Blood-brain barrier-related genes. GLUT-1 and PGP were the highest for the iNPC group, lowest for the MSC group. The iECs differentiation protocol used in this study generate the comment ECs, not the brain microvascular ECs. *indicates *p* < 0.05.

**
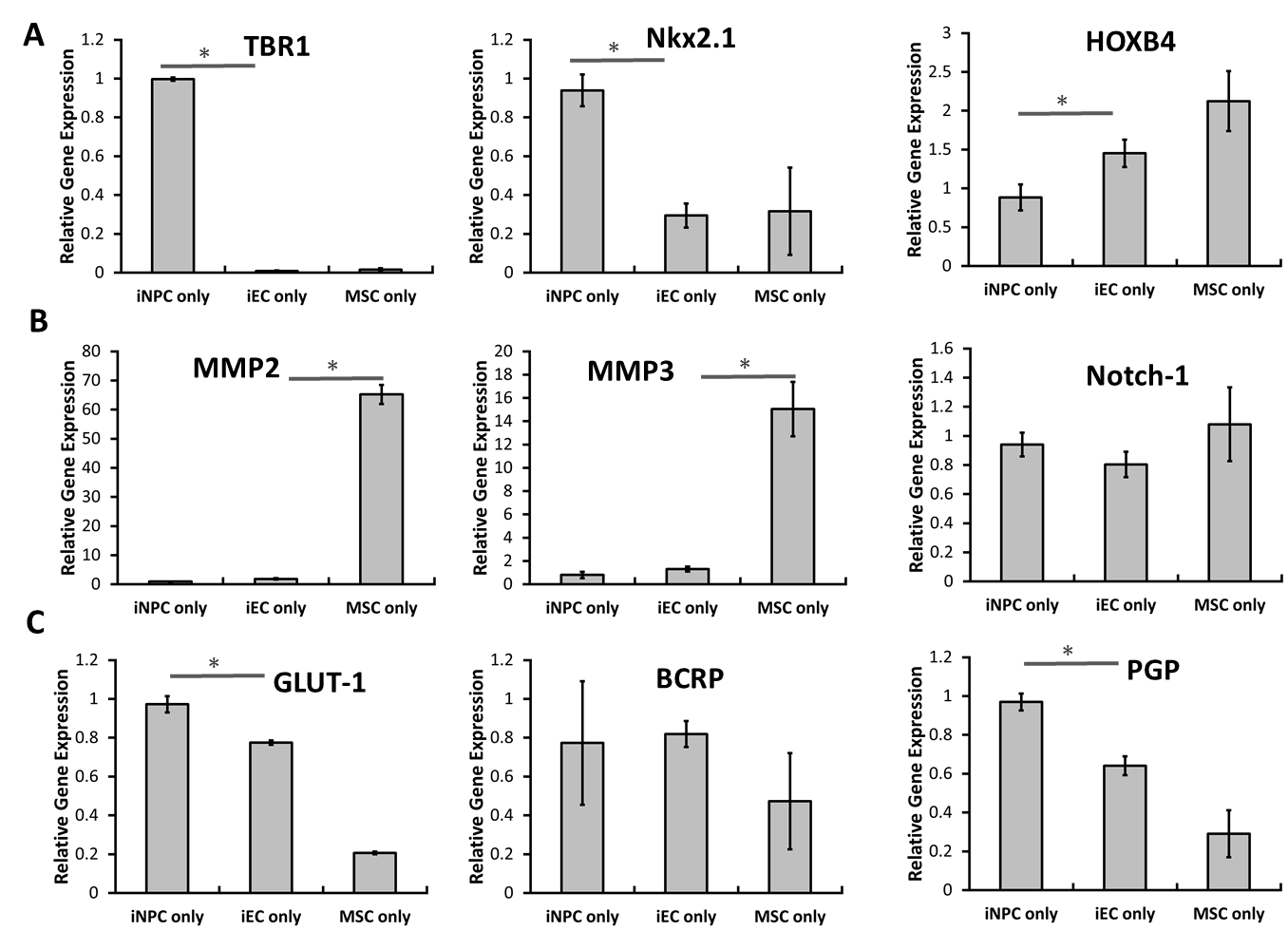
**

**Supplementary Figure S19. Investigation of neurogenesis of hybrid iNPC-MSC spheroids on 2D surface with iEC layer.** (Ai) Day 21 hybrid iNPC-MSC spheroids were replated over per-cultured iECs for three days and immunocytochemistry was performed for neural marker β-tubulin III. Scale bar: 200 μm. (Bi) Phase contrast images of axon outgrowth of day 21 hybrid iNPC-MSC spheroids replated over per-coated endothelial cells. Scale bar: 200 μm. Average neurite lengths were analyzed and shown on (Aii) and (Bii), respectively. *indicates *p* < 0.05 for the different test conditions.


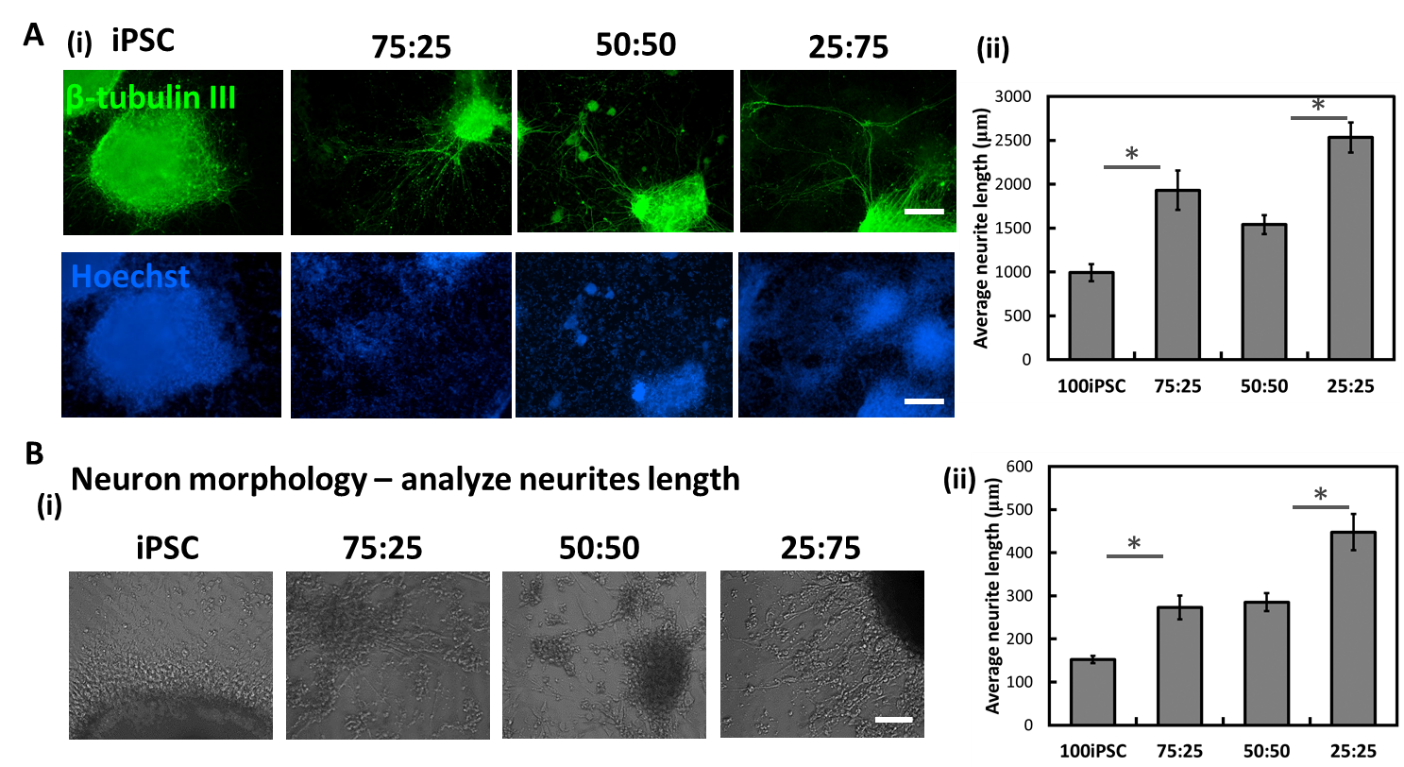


**Supplementary Figure S20. Effects of ROCKi Y27632, Geltrex, and HA on neurogenesis of hybrid spheroids.** Day 21 hybrid spheroids embedded in GelTrex (5%) or treated with ROCKi Y27632 (10 μM) were replated on 1% GelTrex-coated surface for three days. (A) Neurite and axonal extension indicated by β-tubulin III expression. Scale bar: 100 μm. (B) Phase contrast images of axon outgrowth of day 21 spheroids. Scale bar: 200 μm. (C) Representative fluorescent images for day 39 spheroids: neuron marker β-tubulin III (green), motor neuron progenitor marker Islet-1 (red), GABAergic neuron marker (GABA, red), glutamatergic neuron marker (Glutamate, red), cortical neuron marker (TBR1, red), pre-synaptic marker synapsin I (red) and post-synaptic marker PSD95 (red). Hoechst: blue. Scale bar: 100 μm.

**
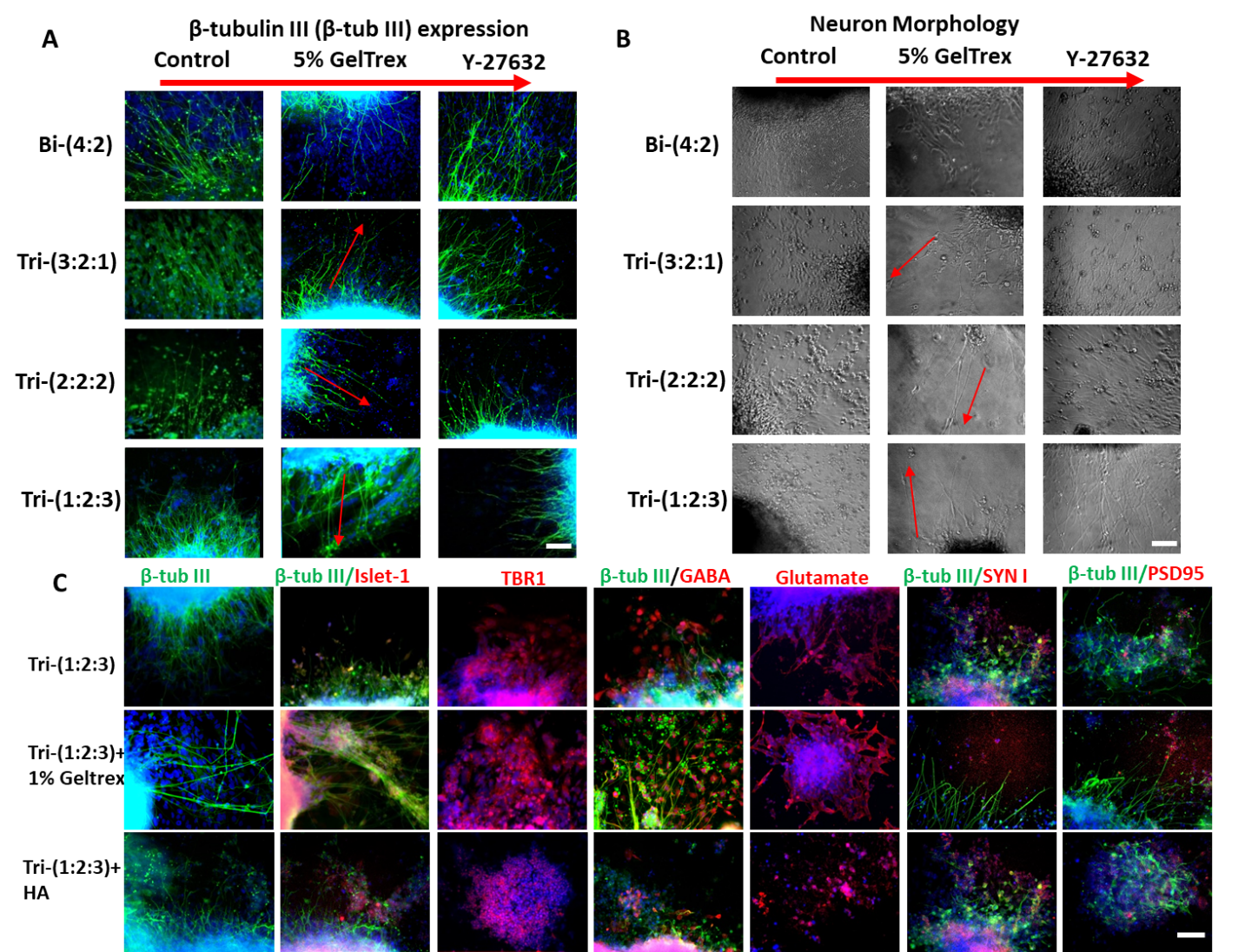
**

**Supplementary Figure S21. Effects of AMD3100 treatment on MTT activity of hybrid spheroids.** The day 24 (after three cell types were put together) hybrid spheroids were measured for MTT activity assay after AMD3100 treatment. *indicates *p* < 0.05 for the different test conditions.


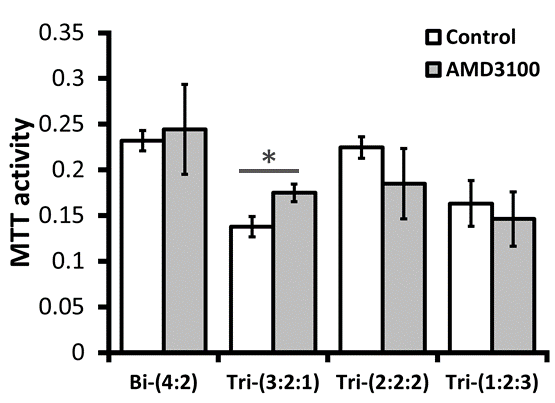


**Supplementary Table S1. A list of antibodies.**

| **Cells** | **Primary Antibody** | **Origin/ Isotype** | **Supplier/ Cat#** | **Dilution** |
| --- | --- | --- | --- | --- |
| Neural cells | Nestin | Rabbit IgG | Sigma, N5413 | 1:100 |
|  | FOXG1 | Rabbit IgG | ThermoFisher, PA5-26794 | 1:100 |
|  | TBR1 (layer VI) | Rabbit IgG | ABCAM, ab31940 | 1:200 |
|  | SATB2 (layer IV) | Mouse IgG_1_ | ABCAM, ab51502 | 1:5 |
|  | BRN2 (layer III) | Goat IgG | Santa Cruz, sc-6029 | 1:200 |
|  | β-tubulin III | Mouse IgG_1_ | Millipore, MAB1637 | 1:200 |
|  | MAP-2 | Rabbit IgG | ABCAM, ab32454 | 1:200 |
|  | ISL1 | Rabbit IgG | Millipore, ab4326 | 1:300 |
|  | HOXB4 | Rabbit IgG | ABCAM, ab76093 | 1:200 |
|  | Glutamate | Rabbit IgG | Sigma, G6642 | 1:1000 |
|  | GABA | Rabbit IgG | Sigma, A2052 | 1:1000 |
| Synaptic markers | Synapsin I | Rabbit IgG | Millipore, 574777 | 1:500 |
|  | PSD95 | Rabbit IgG | Life Technologies, 51-6900 | 1:200 |
| Astrocytes | GFAP | Mouse IgG_1_ | Millipore, MAB360 | 1:400 |
|  | S100B | Mouse IgG1 | Santa Cruz, sc-58839 | 1:100 |
|  | Vimentin | Mouse IgM | Santa Cruz, sc-80975 | 1:100 |
|  | Aldolase C | Mouse IgG1 | Santa Cruz, sc-271593 | 1:100 |
| Endothelial cells | KDR | Mouse IgG_1_ | Millipore, 05-554 | 1:100 |
|  | CD31 | Goat polyclonal IgG | Santa Cruz, sc-1506 | 1:200 |
|  | VE-cadherin | Goat polyclonal IgG | Santa Cruz, sc-6458 | 1:200 |
|  | ZO-1 | Mouse IgG_1_ | Life Technologies, 33-9100 | 1:100 |
| Cell-cell adhesion | E-cadherin | Mouse-IgG_1_ | Millipore; MABT26 | 1:100 |
| Extracellular matrix | Collagen IV | Rabbit IgG | ABCAM, ab6586 | 1:200 |
|  | Laminin | Rabbit IgG | ABCAM, ab11575 | 1:200 |
|  | CSPG | Mouse IgM | Life Technologies, MA1-83055 | 1:100 |
|  | Hyaluronic acid | Sheep IgG | Life Technologies, PA1-85561 | 1:50 |
| Proliferation | BrdU | Mouse IgG_1_ | Life Technologies, 03-3900 | 1:200 |
| Secondary | Alexa 488, goat anti-mouse IgG_1_ | - | Life Technologies, A-21121 | 1:200 |
|  | Alexa 488, goat anti-rabbit IgG | - | Life Technologies, A-11034 | 1:200 |
|  | Alexa 488, goat anti-mouse IgM | - | Life Technologies, A-21042 | 1:200 |
|  | Alexa 594, donkey anti-sheep IgG | - | Life Technologies, A-11016 | 1:400 |
|  | Alexa 594, goat anti-rabbit IgG | - | Life Technologies, A-11012 | 1:400 |
|  | Alexa 594, donkey anti-goat IgG | - | Life Technologies, A-11058 | 1:400 |

**Supplementary Table S2. Primer sequence for target genes.**

| Gene | Forward primer 5' to 3' | Reverse primer 5' to 3' |
| --- | --- | --- |
| TBR1 | CCCCCTCGTCTTTCTCTTACC | TAATGTGGAGGCCGAGACTTG |
| HOXB4 | AATTCCTTCTCCAGCTCCAAGA | CCTGGATGCGCAAAGTTCA |
| Nkx2.1 | GAGTCCAGAGCCATGTCAGC | GCATAAAACAGCTTTGGGGTGT |
| MMP2 | CATCGCTCAGATCCGTGGTG | GCATCAATCTTTTCCGGGAGC |
| MMP3 | CCATCTCTTCCTTCAGGCGT | ATGCCTCTTGGGTATCCAGC |
| Notch-1 | CACTGCTGCCCTCCCCGTTC | TTCAGGTGCCCGATGCCCAG |
| GLUT-1 | AGCAACTGTGTGGTCCCTACG | AAGGTCCGGCCTTTAGTCTCA |
| BCRP | CAGGTGTGCGTCAGAATCATC | TCCAGGAGTGGTCAGATTCCTT |
| PGP | ACCACTCTCCCACCTCCCTTA | TTTAGCTGGGCTGCGTTTACA |
| β-actin | GTACTCCGTGTGGATCGGCG | AAGCATTTGCGGTGGACGATGG |

**References:**

[1] L. Song, A.C. Tsai, X. Yuan, J. Bejoy, S. Sart, T. Ma, Y. Li, Neural differentiation of spheroids derived from human induced pluripotent stem cells-mesenchymal stem cells co-culture, Tissue Engineering Part A 24(11-12) (2018) 915-929.
